# Recent advances in the developmental origin of neuroblastoma: an overview

**DOI:** 10.1186/s13046-022-02281-w

**Published:** 2022-03-11

**Authors:** Mirco Ponzoni, Tiziana Bachetti, Maria Valeria Corrias, Chiara Brignole, Fabio Pastorino, Enzo Calarco, Veronica Bensa, Elena Giusto, Isabella Ceccherini, Patrizia Perri

**Affiliations:** 1 Laboratory of Experimental Therapies in Oncology, IRCCS Istituto Giannina Gaslini, Via G. Gaslini 5, 16147 Genoa, Italy; 2 U.O. Proteomica e Spettrometria di Massa, IRCSS Ospedale Policlinico San Martino, Genoa, Italy; 3 Laboratory of Genetics and Genomics of Rare Diseases, IRCCS Istituto Giannina Gaslini, Genoa, Italy

**Keywords:** Neural crest, Neuroblastoma, Adrenergic, Mesenchymal, Core regulatory circuitries, Transcription factors

## Abstract

Neuroblastoma (NB) is a pediatric tumor that originates from neural crest-derived cells undergoing a defective differentiation due to genomic and epigenetic impairments. Therefore, NB may arise at any final site reached by migrating neural crest cells (NCCs) and their progeny, preferentially in the adrenal medulla or in the para-spinal ganglia.

NB shows a remarkable genetic heterogeneity including several chromosome/gene alterations and deregulated expression of key oncogenes that drive tumor initiation and promote disease progression.

NB substantially contributes to childhood cancer mortality, with a survival rate of only 40% for high-risk patients suffering chemo-resistant relapse. Hence, NB remains a challenge in pediatric oncology and the need of designing new therapies targeted to specific genetic/epigenetic alterations become imperative to improve the outcome of high-risk NB patients with refractory disease or chemo-resistant relapse.

In this review, we give a broad overview of the latest advances that have unraveled the developmental origin of NB and its complex epigenetic landscape.

Single-cell RNA sequencing with spatial transcriptomics and lineage tracing have identified the NCC progeny involved in normal development and in NB oncogenesis, revealing that adrenal NB cells transcriptionally resemble immature neuroblasts or their closest progenitors. The comparison of adrenal NB cells from patients classified into risk subgroups with normal sympatho-adrenal cells has highlighted that tumor phenotype severity correlates with neuroblast differentiation grade.

Transcriptional profiling of NB tumors has identified two cell identities that represent divergent differentiation states, i.e. undifferentiated mesenchymal (MES) and committed adrenergic (ADRN), able to interconvert by epigenetic reprogramming and to confer intra-tumoral heterogeneity and high plasticity to NB.

Chromatin immunoprecipitation sequencing has disclosed the existence of two super-enhancers and their associated transcription factor networks underlying MES and ADRN identities and controlling NB gene expression programs.

The discovery of NB-specific regulatory circuitries driving oncogenic transformation and maintaining the malignant state opens new perspectives on the design of innovative therapies targeted to the genetic and epigenetic determinants of NB. Remodeling the disrupted regulatory networks from a dysregulated expression, which blocks differentiation and enhances proliferation, toward a controlled expression that prompts the most differentiated state may represent a promising therapeutic strategy for NB.

## Neuroblastoma

Neuroblastoma (NB) is a pediatric tumor originating from the developing peripheral sympathetic nervous system, specifically from neural crest (NC)-derived cells undergoing a defective sympathetic neuronal differentiation due to genomic and epigenetic impairments. NB may arise at any final site reached by migrating neural crest cells (NCCs) and their progeny, preferentially in the adrenal medulla (AM) or in the paraspinal ganglia.

NB accounts for about 10% of all pediatric cancers and usually affects children within the first 5 years of life. NB at onset may occur either as localized or metastatic disease and patients are classified in different risk groups defined by several prognostic factors including age at diagnosis, stage, histological features, and molecular alterations. NB substantially contributes to childhood cancer mortality, particularly in high-risk patients suffering chemo-resistant relapse, whose survival rate hardly reaches 40% [1–4].

NB remains a challenge in pediatric oncology and the design of new targeted therapies aimed at interfering with specific genetic/epigenetic alterations represents a beneficial strategy to achieve patient-tailored precision medicine approaches. However, NB is a complex disease showing a remarkable biological and genetic heterogeneity that critically depends on the interaction of several driver and suppressor genes, either coding or non-coding, which act within regulatory networks [5] and in interrelated pathways to cause or modify disease phenotype [6].

From the genetic point of view, NB may present numeric or structural chromosomal alterations such as 1p, 11q, 14q deletions, and 17q gain [3] as well as several gene alterations, all of which correlating with worse prognosis. Specifically, tumors may exhibit *MYCN* amplification [7–9] and germline or somatic mutations of *ALK* [10–13] and *PHOX2B* [14–17], rearrangements at *ATRX* [18] and at *TERT* [19, 20] loci. *MYCN* amplification *ATRX* and *TERT* alterations, which are predominant in high-risk patients, lead to telomere maintenance and in combination with RAS and/or p53 pathway mutations, increase tumor aggressiveness and worsen the prognosis [20]. Moreover, deregulation of gene expression, especially overexpression of *MYCN* [4, 21–24], *ALK* [10–13], *PHOX2B* [25, 26], and *LIN28B* [27, 28], play a key role in initiation and progression of NB by altering the balance between cell proliferation and differentiation [16, 29–31].

Transcriptional and epigenetic analyses have identified phenotypically divergent states of cellular differentiation that are highly influenced by genetic events or epigenetic perturbations occurring during tumorigenesis that remodel the NB regulatory landscapes [32, 33].

Here, we review the most recent studies that have identified NC-derived cell populations relevant to NB oncogenesis and the transcription factor regulatory circuitries that drive oncogenic transformation and maintain the malignant state.

## Neural crest cell fates in physiologic development

NCCs represent a multipotent and migratory cell population in the developing embryo that contributes to the formation of a wide range of tissues, including neurons and ganglia of the sympathetic (or autonomic) nervous system and the AM.

NC-derived cells persist into adulthood and due to their stemness capacity may have important roles not only in tissue regeneration, but also in cancer initiation [34].

Impaired development, differentiation, and migration of NCCs are causative for a class of syndromes, known as neurocristopathies, and drive oncogenesis of multiple pediatric malignancies, including NB, peripheral primitive neuroectodermal tumors, malignant peripheral nerve sheath tumors, craniofacial osteosarcoma, as well as adult malignancies such as melanoma [35].

NCCs have aroused interest for their multipotency features and their extensive migratory capacity since their discovery about 150 years ago [34, 36]. During this long period of time, experimental embryology has made use of a variety of animal models and a plethora of advancing technologies to follow NCC fates, including interspecies grafts, avian chimeras, vital dye labeling, genetic cell labeling via retroviral infection, Cre-loxP systems, in vitro clonal analysis, clonal analysis using multicolor reporters or retroviral infections to integrate sequences encoding barcodes, histological markers and fluorescent proteins for lineage tracing in embryos [34]. Presently, single-cell (sc) spatial transcriptomics, combined with lineage tracing or with genome editing-based lineage reconstruction at the population and clonal levels, represent the most innovative and informative tool to identify transient states and branch points in early cell fate decisions.

Most literature studies on embryonic development are based on the assumption that it is possible to recreate human events leading to organogenesis or to malignancy in animal models, particularly in rodents. NB does not naturally occur in mice and, although numerous studies in mouse models have improved our knowledge about many aspects of biology and genetics of NB, current models do not fully recapitulate the peculiar features of the human disease [37].

NC development was extensively investigated in mouse models mainly by lineage tracing and the fundamental processes and migration routes of NCCs proved to be similar to those in humans [38, 39]. Nevertheless, the most recent studies based on single-cell RNA sequencing (scRNA-seq) in developing mouse and human embryos at different time points and stages of development, particularly the sympatho-adrenal lineage that is related to human NB development, revealed some differentially expressed markers in mouse vs human cells, highlighting important interspecies differences [40–48] (see  paragraph: Sympatho-adrenal cell populations).

On the basis of the latest findings, in the present review we have reconstructed and schematically illustrated in the first three figures the multistep physiologic development of NC-derived lineages, starting from the first phases that give rise to diverging branches toward the differentiation trajectory of the sympatho-adrenal lineage either in mice or in humans. In addition, we reported in two tables the main protein markers encoded by genes specific for each developmental cell type and engaged in a positive interplay within the gene regulatory network controlling NC formation and derivatives, as identified in mice and in humans.

The herein reviewed studies made use of the terms sympathoblast, sympathetic neuroblast or neuroblast as synonyms that identify the same cell population.

### Cell fate decisions from NCCs to sympatho-adrenal progenitors

**Fig. 1:**
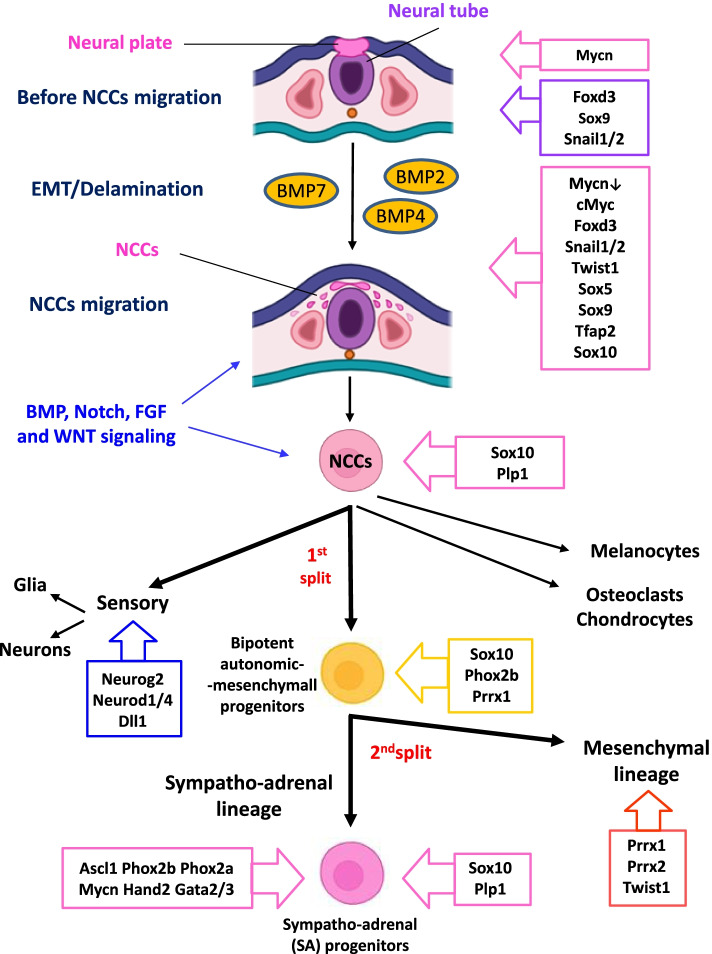
Early stages of physiologic development of NC-derived lineages. Summary of the main processes and gene pathways involved in the early steps of physiologic development of NC-derived lineages emerging from mouse studies and common in mammals. Specifier transcription factors are reported in boxes in hierarchical order. Illustrations has been retrieved from https://app.biorender.com

During the physiological development, NCCs represent a transient population of multipotent stem cells located at the neural plate border before migration (Fig. 1) that, upon inductive signals give rise to increasingly committed cell progeny diverging into different lineages [49].

Distinct inductive signals model the embryonic ectoderm, activate the expression of neural plate border specifier genes and promote the expression of NC specification genes, principally transcription factors (TFs) [49] (Fig. 1, listed in boxes).

The NC specification program expresses a set of regulators belonging to BMP, WNT, Notch and FGF signaling pathways that coordinate the expression of specifier TFs, which in turn activate the Epithelial-to-Mesenchymal Transition (EMT) machinery and the delamination, supplying NCCs with migratory properties [49] (Fig. 1).

For these sequential events to proceed correctly, NCCs constantly change their responsiveness to factors they encounter, i.e. BMP, WNT, Notch and FGF signaling [49–54] and give rise to specific progenitors diverging into several lineages that differentiate into a myriad of cell types: nervous sensory lineage, mesenchymal lineage, sympatho-adrenal lineage and melanocytes, osteoclast and chondrocyte lineages [35, 54–56] (Fig. 1).

The dorsal aorta acts as a morphogenetic signaling center that coordinates NCC migration and initiates a molecular cascade that instructs sympatho-adrenal specification and segregation.

BMPs expressed by the dorsal aorta are critical for the production of SDF1 and NRG1, which act as chemo-attractants for early migration [54, 57]. Later, BMP signaling is directly involved in the migration of common early progenitors of the sympatho-adrenal lineage (SA progenitors) that give rise to sympathetic ganglia and AM (Fig. 1) and promotes the sympatho-adrenal lineage segregation and the ventral displacement of the AM lineage [54].

One of the most recent scRNA-seq study with spatial transcriptomics and lineage tracing conducted in developing mouse embryos by Soldatov et al. [56] disclosed in detail how and when multipotent progenitors deriving from NCCs choose among multiple downstream fates. Multipotent progenitors are cells exhibiting transcriptional or epigenetic heterogeneity that is related to early cell fate decisions and transient states assumed by cells during developmental progressions [56]. The authors identified a first stable bifurcation from NCCs which separates sensory lineage (characterized by the expression marker Neurog2) from autonomic-mesenchymal branches [56] (Fig. 1). Co-expression of Phox2b and Prrx1 markers revealed the existence of common bipotent progenitors of the autonomic-mesenchymal lineages that maintain a multilineage transcriptional expression pattern (Fig. 1). Subsequently, bipotent progenitors undergo a second fate split which separates mesenchymal differentiation (expression marker Prrx1) from the sympatho-adrenal fate (expression marker Phox2b) [56] (Fig. 1).

The *PHOX2B* gene is considered a master gene of autonomic neuron development as its absence was demonstrated to cause the complete loss of peripheral autonomic neurons due to lack of expression of all the other TFs specific of the sympathetic nervous system [58]. In mouse models, Phox2b appeared in the precursors of sympathetic neurons (Sympathetic Neural Progenitor Cells or SNPCs) [59] as soon as they aggregated on each side of the dorsal aorta, while another early expressed TF, Ascl1 (or Mash1), was initially produced independently of Phox2b. Later on, Ascl1 expression was maintained by Phox2b, as demonstrated by its loss in the absence of Phox2b expression [58, 60]. Both Phox2b and Ascl1 gave origin to a downstream hierarchical cascade of TFs represented by Phox2a, Hand2, Gata2–3 (Fig. 1), ending with Dbh and Th proteins, specific of noradrenergic neuronal subtypes [31, 58, 60, 61].

During fetal central nervous system development, the *MYCN* gene resulted initially expressed at high levels, thus promoting ventral migration of NCCs from the neural plate border [62], then its expression decreased to very low levels in migrating NCC with subsequent re-expression in NC-derived lineages that allows maintenance of neural fate [63, 64]. Specifically, studies in mice revealed that *Mycn* is re-expressed in differentiating sympathetic neurons/ganglia after the onset of Ascl1 and Phox2b expression and is concomitant with Phox2a expression [65], followed by *Hand2*, *Gata2*/3, and *Trk* gene expression [30]. Lastly, *Mycn* was downregulated for terminal differentiation and functionality of sympathetic neurons [64] (Fig. 1).

During nervous system development, the Sox10 protein is transiently expressed and preserves NCCs (Sox10+) from both gliogenic and neurogenic differentiation caused by lineage restriction factors [66]. It was demonstrated that during sympatho-neuronal differentiation, co-expression of Phox2b, Sox10 and Prrx1 could be found in bipotent progenitors (Fig. 1) only in the early phases of neurogenesis [56, 66]. Conversely, Phox2b expression was lost during glial differentiation (Sox10+/Phox2b-) (Table 1).

Neurogenesis at these developmental stages proceeded to sympathetic neurons by sympatho-neuronal differentiation rather than to increased proliferation of differentiated neurons [67]. Also, it was shown that Notch signaling is involved in the control of the ratio of neurons to progenitor/glial cells in the early stages of ganglion formation [67]. The Delta/Notch signaling plays a crucial role in the early phases of neurogenesis, regulating the spatiotemporal patterning of neuronal differentiation. This is characterized by asymmetrical cell division (ACD) of undifferentiated progenitors that produces self-renewing progenitors and dividingneurons that keep a balance between progenitor maintenance and neuron differentiation [67, 68].

ACD is a biological mechanism which produces two unequal daughter cells, one of which resembles a multipotent stem and/or progenitor cell, whereas the other has differentiation potential. Thus, ACD leads to the maintenance of the correct number of both self-renewing (stem or progenitor) and differentiated cells [69]. Similarly to stem cells, and, particularly, to cancer stem cells, human NB cultured cells are characterized by both proliferative and differentiating properties. Deregulation of this balance in cancer stem cells proved to be able to trigger the generation of more cancer stem cells [70].

In NB, it was reported that MYCN overexpression induces symmetric cell division (SCD) for self-renewal, thus increasing the pool of undifferentiated cells, while the decreased expression of MYCN caused ACD, allowing a correct balance between stem cells and differentiated cells [69, 70]. In this light, the central role of MYCN in NB pathogenesis emerges also in the cancer-initiating phases through the regulation of the cell division fate, not only in tumor progression [70].

### Cell fate restrictions from sympatho-adrenal progenitors to committed cells in mice

**Fig. 2:**
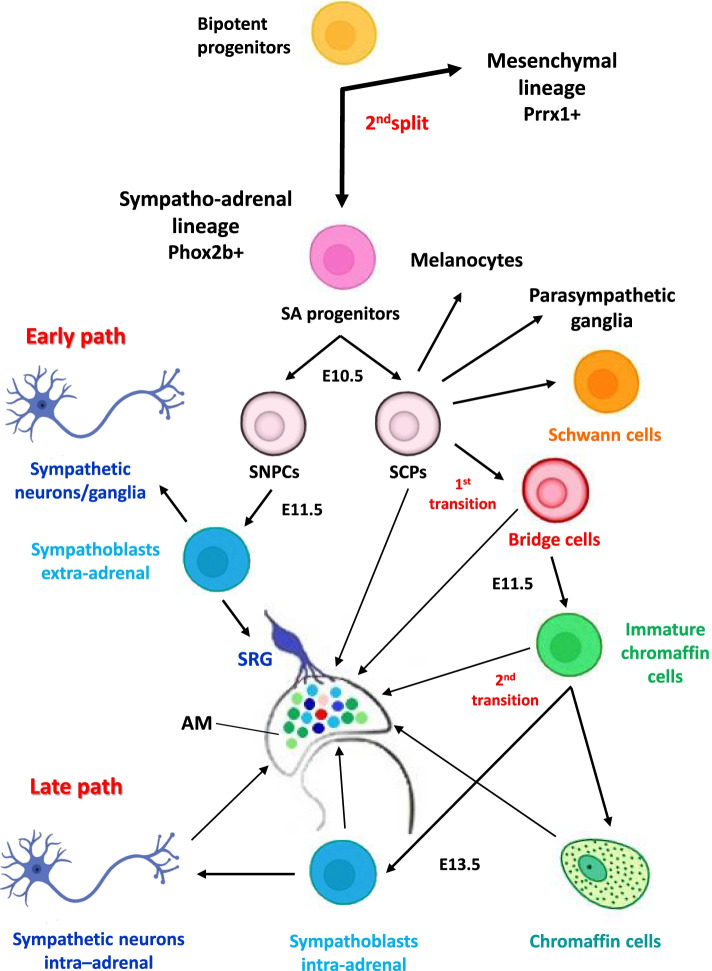
Cell fates and multistep differentiation processes in physiologic development of the sympatho-adrenal lineage in mice. Cell populations and transitions, here illustrated, are based on scRNA-seq studies with spatial transcriptomics and lineage tracing conducted in developing mouse embryos. In the developing AM there is a co-localization of sympathetic and SCP-derived components at different degrees of differentiation, outlined as solid painted circles. Cell type populations involved in physiologic development of the sympatho-adrenal lineage include cell progenitors, transient cells, and cells at different developmental stages toward the final differentiated cell types. Embryonic developmental mouse timeline expressed in days: E10.5, E11.5, E13.5. Basic cell icons have been retrieved from https://app.biorender.com and modified by Adobe Photoshop CC 2019

Sympathetic and adrenergic lineages proved to diverge at an early stage during mouse embryonic development [42] from sympatho-adrenal progenitors [54]. At E10.5 they could be distinguished: (i) committed SNPCs [59] that give rise to differentiated sympathetic neurons and ganglia and, the suprarenal sympathetic ganglion (SRG) (Fig. 2, Early path); (ii) Schwann Cell Precursors (SCPs) that, though committed, still maintain multipotency features and may directly generate glial cells or the chromaffin cells via a transient population of bridge cells [42] (Fig. 2).

The separation of the two branches towards neuronal or glial differentiation was identified by the different transcriptional signatures that include early neuronal markers (Phox2b, Ascl1) in SNPCs [59] or early glial markers (Plp1, Sox10) in SCPs [42] (Fig. 2, Table 1).

**Table 1:** Specifier markers characterizing mouse sympatho-adrenal cell types

MOUSE SYMPATHO-ADRENAL CELL TYPES	MARKERS
**SNPCs**	**Ret Phox2b Ascl1 Mycn Hand2 Gata3** Sox10- Plp1-
**Sympathoblasts extra-adrenal**	**Phox2b Mycn Th Dbh Ret Cart Cartpt** Sox10- Plp1- Sgc10- Ascl1 -
**Sympathetic neurons/ganglia/SRG**	**Ret Scg10 Cart Ascl1 Phox2b Th Dbh** Sox10- Plp1-
**SCPs**	**Sox10 Plp1 Ascl1 Phox2b** Ret-
**Schwann cells**	**Sox10 Plp1 Gfap** Phox2b-
**Bridge cells**	**Sox10 Plp1 Chga Penk Htr3a Ascl1 Phox2b** Th- Ret-
**Immature chromaffin cells**	**Chga Chgb Penk Th Dbh** Sox10- Plp1- Ret-
**Chromaffin cells**	**Chga Penk Ascl1 Phox2b Th Dbh** Sox10- Plp1- Ret-
**Sympathoblasts intra-adrenal**	**Ret Cart Cartpt Phox2b Th Alk Hmx1 Prph** Sox10- Plp1-
**Sympathetic neurons intra-adrenal**	**Ret Cart Ascl1 Phox2b Th** Sox10- Plp1-

Specifier protein markers expressed in each cell population are typed in bold, whereas not expressed proteins are indicated with a - symbol

Thanks to genetic cell lineage tracing of Ascl1, Sox10, Plp1, Ret and other markers, Furlan and co-workers [42] revealed that sympathetic and adrenergic lineages diverge very early during mouse embryonic development (E10.5) (Fig. 2; Table 1). Particularly, Ascl1, which is expressed by both sympathetic and chromaffin cells but at different time points, was helpful in the observation of lineage separation events, revealing that at E11.5 the lineages of sympathetic neurons and chromaffin cells are largely separated (Fig. 2; Table 1).

The authors discovered that chromaffin cells of the AM arise from SCPs and, consistently, genetic ablation of SCPs resulted in marked depletion of chromaffin cells. Genetic ablation of the preganglionic nerve, on which SCPs migrate, similarly led to marked deficiencies of chromaffin cells. They provided evidence that most chromaffin cells arise from SCPs migrating on SRG, which innervates the AM (Fig. 2), and that preganglionic nerves are necessary for AM assembly, highlighting the importance of peripheral nerves as a stem cell niche and migration routes for progenitors essential for neuroendocrine development [42]. SCPs detaching from nerves also generate neuroendocrine cells, para-sympathetic neurons, mature Schwann cells, melanocytes, endoneural fibroblasts, and other cell types [71].

This research revealed a complex gene regulatory mechanism that controls differentiation of SCPs to chromaffin cells. First, SCPs were shown to enter a gene expression program of a transient cellular state (bridge cells) that forms a continuous bridge between SCP and chromaffin clusters, capturing the transcriptional transition between SCPs and chromaffin cells. Subsequently, chromaffin cell gene networks proved to suppress glial gene programs and advance cells into the chromaffin cell differentiation program [42] (Fig. 2; Table 1).

Thanks to numerous lineage-traced clones which appeared unique for SRG and the medulla, this study supports the idea that chromaffin cells in the AM and sympathoblasts in extra-adrenal ganglia derive from different NC and post-NC sub-lineages [42].

In the attempt to provide new insights into the NBs originating in the AM, other recent studies revealed details on the main involved cell types.

One study based on scRNA-seq and functional annotations provided an atlas of the developing mouse adrenal gland (AG), identifying five main cell cluster groups involved (medulla, cortex, endothelial, stroma, and immune clusters), among them the medulla cluster was further divided into seven clusters for comparison with adrenal NB sc-transcriptomes [72] (see paragraph: From physiological development to neuroblastoma origin).

More recently, the development of sympathetic neurons and ganglia of head and trunk in mice, starting from committed SNPCs, was proven to follow an early differentiation pathway [45] (Fig. 2, Early path). Conversely, after a first transition from SCPs to bridge cells, these cells were shown to generate immature chromaffin cells that in turn may undergo complete differentiation or may be subjected to a second transition from chromaffin immature state to intra-adrenal sympathoblast differentiation [45] (Fig. 2, Late path) (see next paragraph).

### Cell fate restrictions from sympatho-adrenal progenitors to committed cells in humans

In the last few years, further studies aimed at elucidating whether the human developing sympatho-adrenal cell populations resemble the molecular features and cell differentiation states of NBs originating in the AM, provided new insights into the main developmental cell types involved also in humans.

These sc-based transcriptome analyses defined more in depth the cell lineage trajectories involved in the development of human AM and results were validated by immunohistochemical staining with specific markers [43–45].

While morphological and hormonal differences in the adrenal cortex were found between humans and mice, few differences were observed in the AM [73]. However, differently from the short time window of the sympatho-adrenal development in mice, in humans the transition from SCP to the complete sympatho-adrenal differentiation persists over several weeks and involves a wider range of transitory cell states.

Dong et al. [43] used scRNA-seq to generate transcriptomes of both human embryonic and fetal adrenals and identified three biologically distinct cell types, namely SCPs, chromaffin cells, and sympathoblasts. They demonstrated that the first phase of AM organogenesis involves an expansion of SCPs and the differentiation of several quiescent chromaffin cells, while in the second phase a substantial number of cycling chromaffin cells expand in numbers by proliferation.

**Fig. 3:**
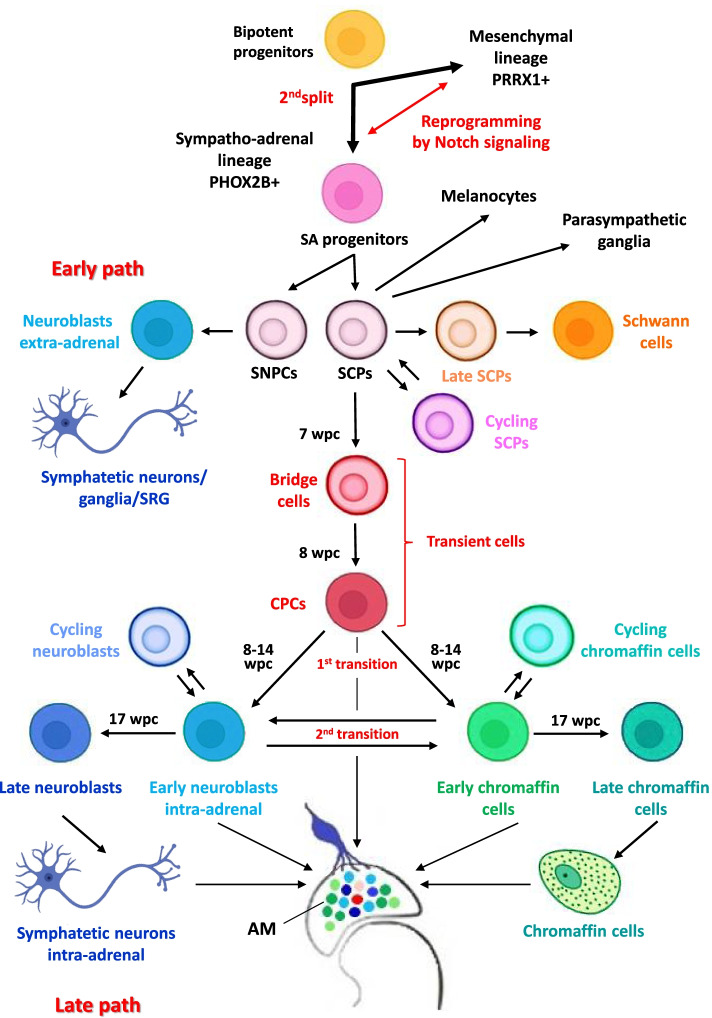
Cell fates and multistep differentiation processes in physiologic development of the sympatho-adrenal lineage in humans. Cell type populations involved in physiologic development of the sympatho-adrenal lineage, here illustrated, include: bipotent cell progenitors, sympatho-adrenal (SA) progenitors, and SNPCs, as emerged from lineage tracing in mouse embryos; SCPs, transient cells and cells at different developmental stages toward the final differentiated state, based on scRNA-seq studies with spatial transcriptomics conducted in human embryos. In the developing adrenal medulla (AM) there is a co-localization of SCP-derived components, sympathetic and chromaffin at different degrees of differentiation, which are outlined as solid painted circles. Developmental human timeline expressed as wpc: weeks post conception. Basic cell icons have been retrieved from https://app.biorender.com and next modified by Adobe Photoshop CC 2019

Jansky et al. [44] identified the main cell types of the sympatho-adrenal lineage in humans. From 7 to 17 weeks post conception (wpc), SCPs (SOX10+, PLP1+, ERBB3+), chromaffin cells (DBH+, TH+, CHGA+) and neuroblasts (ISL1+, ALK+) co-clustered in the AM anlage (Fig. 3; Table 2). Based on transcriptional diversity, the authors estimated the differentiation potential of AM cell types and discriminated transient populations. Transcriptome selection and re-embedding of SCPs, chromaffin cells, and neuroblasts by additional markers at different time points highlighted an early (7–8 wpc) bridge population (ERBB4+, ASCL1+) connecting SCPs and chromaffin cells (Fig. 3; Table 2). A new cell population was detected at 8 wpc, named Connecting Progenitor Cells (CPCs), that spans the transcriptional space between bridge and chromaffin/neuroblast populations (Fig. 3). CPCs diverged into chromaffin and neuroblast populations at 14 wpc. Notably, neuroblasts and SCPs were found to harbor distinct subpopulations of cycling cells, marked by the expression of *MKI67* and *TOP2A*, not present in chromaffin or bridge cells, which indicates that SCPs and neuroblasts have a high proliferative capacity [44] (Fig. 3; Table 2). At later time points (17 wpc), neuroblasts showed increased expression of the differentiation markers and IL7, whereas ALK decreased (Fig. 3, Table 2). Using single-molecule RNA FISH (Fluorescent In Situ Hybridization) to analyze the localization of medullary cell types, this study revealed that single SCPs were located inside nests of neuroblasts and in the close vicinity of small groups of chromaffin cells surrounding these nests, which supports the common derivation of neuroblasts and chromaffin cells from SCPs and SCP-derived transient cells (Fig. 3). Based on transcriptional diversity of cell populations, the authors also identified the starting point of the differentiation trajectory. SCPs showed the highest differentiation potential and, therefore, were placed at the root of the AM differentiation hierarchy. The bridge population also displayed high stemness and subsequently bifurcate, indicating that NC-derived SCPs are common progenitors. Bifurcation of the trajectories at CPCs into neuroblasts and chromaffin cells and differentiation of SCPs into late SCPs were further supported by RNA velocity. Then, the authors identified genes significantly associated with these trajectories, especially TFs that mark cell type transitions. While *PHOX2B* was expressed during SCP to bridge transition, *HAND2* was expressed in the bridge population and *GATA3* later on during bridge to neuroblast transition (Fig. 3¸ Table 2). Expression of the cell cycle inhibitor *CDKN1C* in chromaffin cells and of the activator of cell cycle transition *CCND1* in neuroblasts underlined the diverging cell cycle activities observed in chromaffin cells and in neuroblasts (Table 2). Moreover, chromaffin differentiation resulted to be associated with the activity of many members of the Jun/Fos family of transcription factors, while GATA3, SOX11 and TFAP2B showed high transcriptional activities in neuroblasts [44]. Furthermore, Kameneva et al. [45] examined cell type heterogeneity and dynamics of the developing human and mouse sympatho-adrenal regions, identifying and characterizing new lineage connections that might have cell fates and transitions relevant to NB. This work led to the important and unexpected discovery that, differently from the development of sympathetic neurons and ganglia of head and trunk, during development of the sympatho-adrenal system, intra-adrenal sympathetic neuroblasts arise from SCPs [45]. In addition, the authors demonstrated that these immature sympathoblasts can transition into local neuroendocrine chromaffin cells [45] (Fig. 3). Starting from dissected human embryonic AGs with surrounding tissue from 6 to 14 wpc, sc-transcriptomic analysis revealed the expression of many genes and TFs associated with specific cell types of different maturity: *SOX10*, *PLP1*, *FOXD3* with SCPs, *PRPH*, *STMN2*, *ISL1*, *TH* with sympathetic neuroblasts and *CHGA*, *PNMT*, *PENK*, *TH* with chromaffin cells (Fig. 3, Table 2). Re-analysis of cells, omitting annotated cell cycle genes, made it possible to elucidate the sympatho-adrenal cell fate transitions at higher resolution and embedding results showed that SCPs in human embryos connect to sympathoblasts and chromaffin cells through a fork-like transition (Fig. 3, 1^st^ transition; Table 2). RNA velocity analysis of both embeddings indicated a transition from SCPs into sympathoblasts and chromaffin cells and bioinformatic predictions were experimentally validated by immunohistochemistry. The histological examination of SCPs, sympathoblasts and chromaffin cells in sections of human embryonic AGs at the same developmental stages also showed that chromaffin cells were diffuse chain-like conglomerates mixed with cortical cells and large sympathetic ganglia-like proliferative structures. The joint embedding of human SCPs, sympathoblasts and chromaffin cells revealed a second transition (Fig. 3, 2^nd^ transition) between sympathoblasts and chromaffin cells, showing a direct overlap of the two expression programs and a transient expression patterns. The RNA velocity analysis suggested that the predominant direction of the second transition was from sympathoblasts to chromaffin cells and bioinformatic analysis also supported a reverse direction of transition [45] (Fig. 3, 2^nd^ transition). In the light of these newly observed transitions in humans, not previously detected in mice [42], the authors sought some clues to clarify this inconsistency between the two species [45]. Previous lineage-tracing experiments demonstrated that the vast majority of mouse sympathoblasts in SRG and the sympathetic chain originate directly from migratory NC-derived cells without transition via the SCP state [42] (Fig. 2, Early path). To test the hypothesis that sympathoblast subtypes in intra- and extra-adrenal populations may have different developmental fates and that there might be an uncharacterized population of SCP-derived sympathoblasts also in mice, the authors performed scRNA-seq of lentivirus-based lineage-tracing of NC derivatives in the mouse AG at different embryonic stages. The re-analysis of the differentiation trajectory and RNA velocity has shown that the transition from SCPs to chromaffin cells was consistent with the findings of earlier studies [42]. However, a steady transition between immature chromaffin cells and sympathetic cells was also observed. RNA velocity estimated that the direction of this transition in mice was from chromaffin cells to sympathoblasts and lineage tracing confirmed a model in which chromaffin cells, originating from SCPs, contribute to the sympathetic population via bridge cells and an intermediate immature chromaffin cell state [45] (Fig. 2, Late path). These results confirmed different sympathoblast fates: extra-adrenal sympathoblasts differentiating through an early pathway [45] from SNPCs [59] (Fig. 2, Early path) and intra-adrenal sympathoblasts deriving through a late pathway from SCPs (Fig. 2, late path) [45]. The two sympathoblast lineages were shown to originate from common sympatho-adrenal progenitors [54] which largely split at E10.5 [42] (Fig. 2). Specific marker expression demonstrated that after splitting, committed cells differentiate into sympathetic neurons/ganglia and SRG (Fig. 2, Early path), while SCPs migrate along the nerve tracks towards the AM and give rise to chromaffin cells and intra-adrenal sympathetic neurons through a late pathway via an immature chromaffin state (Fig. 2, Late path) [42]. Therefore, the fraction of sympathoblasts within the AM originates from immature chromaffin cells that derive from SCPs via bridge cells (Htr3a+). According to mouse developmental time, chromaffin cells begin to appear from E11.5, while intra-adrenal sympathoblasts (Cart+) appear later, around E13.5 (Fig. 2, Late path). Despite the difference in origin, no phenotypic distinction among ganglia-like structures inside the AM was evident and no major differences in RNA profiles were detected between extra- and intra-adrenal sympathoblasts, in either humans or mice [45].

**Table 2:** Specifier markers characterizing human sympatho-adrenal cell types

HUMAN SYMPATHO-ADRENAL CELL TYPES	MARKERS
**SNPCs**	**RET PHOX2B ASCL1 HAND2 GATA3** SOX10- PLP1-
**Sympathetic neuroblasts extra-adrenal**	**RET PHOX2B TH** SOX10- PLP1- SGC10-
**Sympathetic neurons/ganglia/SRG**	**RET SCG10 ASCL1 PHOX2B TH DBH** SOX10- PLP1-
**SCPs**	**SOX10 PLP1 ERBB3 FOXD3 ASCL1 PHOX2B** RET- ISL1- HAND2-
**Cycling SCPs**	**SOX10 PLP1 ERBB3 ASCL1 MKI67 TOP2A** ISL1- HAND2-
**Schwann cells**	**SOX10 PLP1 GFAP** PHOX2B-
**Bridge cells**	**SOX10 PLP1 PHOX2B HAND2 ISL1 ASCL1 ERBB4 VGF MYCN ALK** RET- TH-
**Early chromaffin cells**	**CHGA CHGB PENK TH DBH** SOX10- PLP1- RET-
**Early Neuroblasts intra-adrenal**	**RET ASCL1 PHOX2B HAND2 GATA3 TH ISL1 ALK MEIS2 NPY PRPH NEFM STMN2 SYN3 CCND1** SOX10- PLP1-
**Cycling chromaffin cells**	**CHGA CHGB PENK TOP2A** SOX10- PLP1- RET-
**Cycling neuroblasts**	**PRPH ISL1 RET ASCL1 PHOX2B HAND2 GATA3 MKI67 TOP2A** SOX10- PLP1-
**Late chromaffin cells**	**CARTPT INSM1 CHGA PNMT PENK ASCL1 PHOX2B TH DBH CDKN1C** SOX10- PLP1- RET-
**Late Neuroblasts**	**SYN3 IL7 RET PHOX2B GATA3 PRPH STMN2 ISL1 TH NTRKI GAP43 SOX4 SOX11 TFAP2B** SOX10- PLP1- ALK-

Specifier protein markers expressed in each cell population are typed in bold, whereas not expressed proteins are indicated with a – symbol

In summary, SCPs were shown to generate bridge cells and the newly identified CPCs, spatially placed between bridge cells and the divergent chromaffin and neuroblast populations [44] (Fig. 3). A first fork-like transition was revealed that separates the intra-adrenal neuroblasts (Fig. 3, 1^st^ transition, Late path) from the neuroendocrine chromaffin cells [45] and further steps that lead to the most differentiated late populations [44] (Fig. 3). Moreover, SCPs and neuroblasts proved to harbor subpopulations of cycling cells (Fig. 3, Table 2), indicative of high proliferative capacity [44]. A population of cycling chromaffin cells was also identified in human fetal AGs in a previous study [43] (Fig. 3 and Table 2).

In humans, intra-adrenal sympathoblasts undergo a second transition into local chromaffin cells and, through a reverse direction of transition, chromaffin cells may also convert into sympathoblasts [45] (Fig. 3, 2^nd^ transition).

The discovery of two different pathways of differentiation of extra-adrenal and intra-adrenal sympathoblasts, the common derivation of chromaffin cells and intra-adrenal sympathoblasts from SCPs, and the ability of transition between chromaffin and sympathetic cell states reveales a remarkable complexity of human AM development. Indeed, this prolonged time of transitions in normal development is likely to increase the risk for each cell type to acquire genetic alterations or to undergo epigenetic signal perturbations promoting NB formation at different levels, in any NC-derived cell population with low degrees of differentiation and with enhanced proliferative state.

Finally, in the latest study by Bedoya-Reina et al. [74], snRNA-seq analysis was performed on postnatal human AGs and on NBs of patients belonging to different classes of risk to be compared. Interestingly, transcriptome analysis of postnatal human AG showed the presence of a subtype of TRKB+ cholinergic progenitor population whose transcriptional profile resulted crucial in the comparison with NB-specific populations (see next paragraph).

## From physiological development to neuroblastoma origin

As most of primary NB tumors arise from the AM, investigations on the potential cell population involved in NB origin have mainly been focused on the embryonic adrenal development.

Mature human AM contains mainly chromaffin cells, while intra-adrenal sympathetic neurons are not so abundant. Anatomically, it was shown that sympatho-adrenal differentiation occurs within the AM and within large highly proliferative sympathetic ganglia-like structures inside the developing adrenal cortex [45]. At cellular level, SCPs resulted located inside nests of neuroblasts with small groups of chromaffin cells clustered around, which is consistent with the common origin of intra-adrenal neuroblasts and chromaffin cells from SCPs and SCP-derived transient cells [44].

To identify which cell populations involved in AM development may be preferentially subjected to tumor transformation during the development and differentiation of sympatho-adrenal lineage, sc-transcriptomes of human developing sympatho-adrenal tissues and adrenal NB tumors were compared.

Because of the limited tumor samples analyzed by scRNA-seq, all results from these studies have been validated by tracking developmental cell-type-related signatures in bulk RNA-seq datasets (in-house or available into GEO database), to identify which signatures may be shared by patients stratified by age, stage of disease, presence of genetic alterations or prognosis. This strategy made it possible to expand RNA-seq information into broader clinical records.

These scRNA-seq studies highlighted the involvement of specific cell types in NB oncogenesis and the developmental plasticity maintained by tumor cells.

Transcriptional and epigenetic profiling also revealed the remarkable intra-tumoral heterogeneity and the epigenetic remodeling of regulatory landscapes in NB. Most NBs included different types of tumor cells with divergent gene expression signatures: an adrenergic-type holding traits of cells committed to the sympatho-adrenal lineage (termed ADRN) and an undifferentiated mesenchymal-type exhibiting features of NC-derived precursors (termed MES) [32, 75]. These cell identities were able to interconvert and resemble cells from different lineage differentiation stages through a mechanism of epigenetic reprogramming [33, 76].

Moreover, Chromatin immunoprecipitation sequencing (ChIP-seq) studies also disclosed the fundamental role of super-enhancers (SEs) and their association with lineage-specific transcription factors (TFs) to form specific Core Regulatory Circuitries (CRCs) for each cell type that underlie MES and ADRN cell identity states.

These molecular aspects are described in detail in paragraph: Core regulatory circuitries and super-enhancers in neuroblastoma.

### Sympatho-adrenal cell populations

Most primary NB tumors originating in the AM presented transcriptional similarities with the sympatho-adrenal cell populations and an epigenetic ADRN profile.

Sc-transcriptomes from early human embryos and fetal AGs and from adrenal NBs were compared by Dong et al. [43] in a study disclosing that several NB expression meta-programs have a transcriptional profile resembling sympathoblasts or, most prevalently, chromaffin cells. By defining gene signatures for undifferentiated and differentiated chromaffin cells, the authors found that most adrenal NB tumor cells transcriptionally reflect a noradrenergic chromaffin cell type and that an undifferentiated chromaffin cell state is mainly associated with malignancy. This supported the hypothesis that heterogeneity in adrenal NB is due to different degrees of proliferation/differentiation in chromaffin cells [43].

In a subsequent study, Hanemaaijer et al. [72] reconstructed an atlas of the developing mouse AG at a single-cell level and identified five main cell cluster groups (medulla, cortex, endothelial, stroma, and immune clusters). The medulla group was further divided into seven clusters, including the SCP and the neuroblast clusters [72]. Data were annotated using expression correlation to the MouseRNAseqData reference dataset (358 bulk RNA samples). The neuroblast cluster resulted the most relevant to NB comparison [72] and identified highly cycling cells that share markers with sympathoblasts, as previously described [42]. When compared with NB cell signatures of patients at different disease stages, present in the TARGET NB RNA-seq dataset (172 bulk RNA samples) and in the NB SEQC RNA-seq dataset (498 bulk RNA samples), the signature of the medullary SCP cluster turned out to be associated with NB phenotype severity and made it possible to group NB patients based on disease phenotype [72]. Specifically, the SCP signature score inversely correlated with *ALK* and *MYCN* expression, that is associated with poor prognosis, while a high SCP signature score correlated with better overall survival rates. This study also showed that healthy neuroblasts express high levels of MycN and Alk, implying that their loci are more open and susceptible to gene amplifications or mutations [72].

In another study, Kildisiute et al. [77] compared sc-transcriptomes from human fetal AG and NB, revealing that NB cancer cells resemble the fetal sympathoblast state [77]. These findings were also validated in a large number of NB bulk transcriptomes from the TARGET RNA-seq and from the SEQC datasets by integrating canonical features of the NB genome with transcriptional signals. However, the transcriptional similarity between NB cancer cells and sympathoblasts emerged as a common feature regardless of cell cluster diversity, individual patients, and clinical features [77].

In a more recent study, Jansky et al. [44] elucidated the developmental programs and cell types resembling NB originating in the human AM. Developing human AM samples from seven developmental time points and NB specimens from patients belonging to broad clinical NB subgroups were analyzed by droplet-based single nucleus RNA-seq (snRNA-seq) to discriminate malignant from nonmalignant cells and data were validated in NB bulk transcriptomes from the TARGET and from the SEQC RNA-seq datasets [44]. First, cells were clustered for expression of known marker genes of infiltrating normal cell types like *PTPRC*, *PTPRB* and *COL1A1* and for further analysis only malignant cells harboring copy number alterations and lacking expression of normal cell type markers were kept. Differently from results obtained by Dong et al. [43], adrenergic markers like CARTPT and INSM1 were found expressed in chromaffin cells, while the neuronal markers *NPY*, *PRPH*, *NTRK1* and *ISL1* were clearly expressed in neuroblasts (Table 2). To support a correct cell type annotation, the authors evaluated the expression of many other sympathetic neuronal marker genes in the neuroblast cluster, including *NEFM*, *STMN2*, *SYN3* and *ALK* (Table 2). Moreover, they found that the well-established chromaffin marker *PNMT* was expressed in a subpopulation of mature chromaffin cells [44].

After determining the transcriptional similarity between malignant NB cells and normal developing AM cell populations, adrenal neuroblasts with high similarity scores were clearly shown to be the best match to NB cells. Then, the authors tried to match the features of normal developmental cell populations to those of the NB risk subgroups defined by genetic alterations: *MYCN*-amplified high-risk tumors, *MYCN*-nonamplified high-risk tumors (*TERT*-rearranged or harboring telomere lengthening), and intermediate/low-risk tumors (lacking telomere maintenance) [9, 20, 78].

*MYCN*-amplified NB cells turned out to be the most similar to normal early neuroblasts, with only few cells matching late neuroblasts, while the proportion of cells similar to late neuroblasts was higher in *MYCN*-nonamplified high-risk tumors and among low-risk NB cells. These data suggested that low-risk tumors arise at a later time point during development or that a higher degree of dedifferentiation is induced in high-risk NBs, especially, in *MYCN*-amplified tumors [44]. The comparison between normal and NB cells at the single-cell level in the diffusion map embedding [79] showed a continuous flow of cells across the two cell types, thereby confirming the neuroblast identity of NB tumor cells [44]. Within the neuroblast population, low-risk tumors were comparable to more differentiated neuroblasts, while high-risk cases tumors mapped closer to the beginning of the neuroblast differentiation trajectory [44].

Developmental trajectories, spanning the space from SCPs via bridge cells and CPCs to neuroblasts and late neuroblasts (Fig. 3), were also traced in the distinct genetic and epigenetic NB subtypes. Expression analysis also determined cell identity genes whose enhancers, via rearrangements/translocations, were found to activate oncogenes such as *MYCN*, *MYC,* and *TERT* [44] through a mechanism of enhancer hijacking that juxtaposes ectopic enhancers or SEs controlled by a specific CRC to the gene transcriptional unit [80, 81]. Remarkably, *MARCH11*, *NPY*, *EBF1*, *HAND2*, *ALK*, *CCND1* and *EXOC4* genes, all involved in enhancer hijacking events in NB, showed the highest expression in neuroblast trajectory cells, suggesting that the neuroblast lineage is specifically at risk of acquiring genetic alterations promoting neuroblastoma formation [44].

To further discriminate NB cells from normal neuroblasts, a differential expression analysis of single NB cells clustered by subtype and their closest matching to normal cell types was performed [44]. It emerged that NB cells of high-risk tumors matched early neuroblasts, while low-risk tumors showed high similarity with their normal counterparts, the late neuroblasts. In low-risk NBs, the expression of developmental genes that define the most differentiated normal neuroblast state, such as *PRPH*, *SYN3*, *GAP43*, *NTRK1,* and *SOX4*, showed the highest expression. In contrast, markers of less differentiated neuroblasts like *ALK* and MEIS2 were expressed at higher levels in high-risk NBs [44].

Evaluation of AM transcription factor activities in NB subgroups showed low-risk tumors harboring the highest levels of TFAP2B, which is highly expressed in normal neuroblasts, while *MYCN*-amplified cells were regulated by MYCN, showing a pronounced oncogenic MYCN signature and a reduced normal neuroblast signature. An inducible *MYCN* knockdown was adopted to evaluate whether MYCN itself could suppress differentiation in *MYCN*-amplified cells. Indeed, NB cells with reduced expression of *MYCN* had higher expression of neuroblast- and late neuroblast-specific genes and showed repression of cell cycle-related genes, indicating that elevated MYCN causes dedifferentiation and proliferative activation. On the other hand, activation of TFAP2B, whose expression is abrogated in high-risk NBs, was able to restore differentiation signatures [44].

These results showed that NBs are transcriptionally similar to developing adrenal neuroblasts and that the differentiation state of NBs along the normal neuroblast differentiation trajectory is associated with prognosis. Low-risk NBs closely resemble the normal committed neuroblast population and reveal the highest degree of differentiation. In contrast, *MYCN*-amplified NBs and NBs with mesenchymal features (see below) were the most undifferentiated subtypes, containing tumor cells with features of early neuroblasts and bridge cells, holding the highest malignant potential [44].

In another recent scRNA-seq study, Kameneva et al. [45] compared developmental cell populations of human AM and other sympatho-adrenal regions with the heterogeneous cell types observed in NB. The authors carried out joint analyses of their data on sympatho-adrenal samples and of scRNA-seq data on additional NB biopsies from patients described in other studies [43, 82].

Results revealed that most adrenergic tumor cells had clear similarity to embryonic sympathoblasts, with a fraction of tumor cells co-aligning with chromaffin cells, mesenchymal cells aligning with the embryonic mesenchymal populations, and Schwann components aligning with the SCP population [45]. The authors also analyzed bulk RNA-seq data from a large cohort of patients from the SEQC NB dataset with different adrenal NB subtypes and gene expression signatures of embryonic SCPs, chromaffin cells, and proliferative and mature sympathoblasts to point out possible associations with patient survival. The lowest survival rate was associated with the transcriptional signature of proliferative sympathoblasts. This association held regardless of the presence or absence of *MYCN* amplification in tumors and was independent of cell cycle genes. The SCP and mature sympathoblast signatures were associated with better prognosis in non-*MYCN*-amplified tumors, whereas the embryonic chromaffin cell signature was associated with poor prognosis, suggesting the possible existence of immature chromaffin-like NB cell subtypes. Finally, *MYCN* amplification in tumors were found to worsen the prognosis of patients with mature sympathoblast signature NB, while the SCP signature always remained correlated with positive disease outcome [45].

It also emerged that the SCP population aligned with the Schwann component of tumor samples [45, 72] and a fraction of tumor cells co-aligned with chromaffin cells [45]. This is consistent with the microscopic morphology of NB cells that is composed of a population of neuroblasts at different degrees of differentiation and a variable number of normal Schwann cells [3, 4], depending on the histopathologic features [83, 84] of each single case.

All the above studies highlight the role of early sympathetic cells or their immediate progenitors in the initiation of NB tumor transformation, particularly in the adrenal localization, where crucial cell fate decision processes occur. The normal development of the peripheral sympathetic nervous system involves many different NC-derived precursors and committed cells that populate various final sites along the routes of migrating NCCs, where they undergo the complete differentiation process. Thus, NB may originate from any of these sites and, likewise adrenal neuroblasts, genetic and epigenetic impairments may affect extra-adrenal neuroblasts committed to the sympathetic chain promoting NB development in para-spinal ganglia. In this regard, it emerged that different genomic aberrations are involved in adrenal NBs vs NBs arising in para-spinal ganglia. A recent study compared genomic and epigenomic data from TARGET project on primary NBs [18] originating in the AM with those arising from thoracic sympathetic ganglia. This study revealed that adrenal NBs are more likely to harbor structural DNA aberrations including MYCN amplification, whereas thoracic tumors show defects in mitotic checkpoints leading to hyperdiploidy [85]. These findings confirm that NB tumors arising in different sites are heterogeneous entities, rather than subclasses of NB [85].

Noteworthy, we should consider that one study [43] came to different conclusions, showing that most adrenal NB tumor cells transcriptionally reflect a chromaffin cell type and that an undifferentiated chromaffin state is associated with malignancy.

These incongruent results are basically due to different cell type annotations. In this regard, the expression of CARTPT and other markers in human developing cells has been a subject of much debate. Based on the observation that, during mouse development, Cartpt expression is restricted to sympathoblasts [41, 42, 46], Dong et al. [43, 48] attributed CARTPT expression to human sympathoblasts. However, the re-analyses of datasets used by Dong in human embryonic cells [43] and by Furlan in mouse developing cells [42] revealed that some clusters, previously designated as human chromaffin cells, resemble mouse developing sympathoblast clusters, whereas the human sympathoblast clusters are likely to resemble to a large extent the mouse chromaffin cells [40, 47]. Such differences underline the reason why mouse model systems cannot fully recapitulate human development and, consequently, NB oncogenesis.

The studies by Jansky et al. [44] and by Kameneva et al. [45] clearly detected the expression of CARTPT in a cluster of chromaffin cells in humans. However, these authors also demonstrated that certain markers of the sympatho-adrenal lineage are highly dynamic during development, in fact there were identified a CPC transient population [44] and a second transition between sympathoblasts and chromaffin cells [45] (Fig. 3), highlighting transient expression patterns and overlaps of the two expression programs. Therefore, it is possible that NB may arise from other cell states that assume the transcriptional state of sympathoblasts upon malignant transformation.

Dong et al. [43] considered that chromaffin cells and sympathoblasts have distinct spatial locations and that the CARTPT+ population was previously shown to be mainly located in the extra-adrenal sites (sympathetic ganglia and SRG) [43, 48], but not in the AM [41, 46]. On the other hand, the recent identification of intra- and extra-adrenal sympathoblasts showing neither phenotypic nor transcriptional distinction [45] changes the perspective.

To broaden the scRNA-seq information and to find a possible association of specific cell types with NB phenotype severity and patient outcome, sc-transcriptomes of the normal sympatho-adrenal populations were compared also with bulk NB RNA-seq transcriptomes, including a large cohort of samples from patients classified by risk subgroups.

Immature or poorly differentiated neuroblasts matched tumor cells of the high-risk group, especially *MYCN*-amplified NB cells, and were associated with poor prognosis, while mature or highly differentiated neuroblasts matched NB cells of the low-risk group and *MYCN*-nonamplified tumor cells and were associated with good prognosis [44, 45, 72, 86].

The signature of the medullary SCPs and late SCPs were significantly associated with favorable prognosis [45, 72] and, consistently, the SCP signature score was inversely correlated with *ALK* and *MYCN* expression, known to be associated with poor prognosis. Some authors hypothesize that this may be explained by either the presence in good prognosis tumors of healthy SCPs that confer a protective effect [72] or by the presence in these tumors of other cell types, like Schwannian stromal cells, also originating from SCPs [44, 72]. Conversely, embryonic chromaffin cells aligned with tumors with unfavorable outcomes, which is consistent with the existence of immature chromaffin-like NB cell subtypes. Notably, *MYCN* amplification was found to worsen the prognosis of NB patients with mature sympathoblast signature, while the SCP signature always remained correlated with positive outcome [45].

Taken together, these findings show that NB can derive from neuroblasts at different stages of differentiation, and that a low degree of differentiation is associated with high-risk tumors, while a higher degree of differentiation is associated with low-risk tumor. Alternatively, a higher degree of de-differentiation may be induced in high-risk NBs, especially in *MYCN*-amplified cases [44].

### Mesenchymal cell populations

Most adrenal NB tumors analyzed in the study by Jansky et al. [44] were classified as ADRN type, yet the malignant cells of three high-risk tumors showed increased MES signature expression and reduced ADRN signature expression and were termed high-risk NBs with MES features, as previously defined [32].

High-risk NBs with MES features showed similarity to a broader set of normal cell populations including bridge, connecting progenitor and chromaffin cells, in addition to neuroblasts, and were mapped close to the branching point of the chromaffin and neuroblast lineages, within the newly defined CPC population. *ERBB4* and *VGF*, which are characteristic of bridge/connecting progenitor cells, were exclusively expressed in high-risk NBs with MES features. In contrast to low-risk NBs that closely resembled the normal committed neuroblast population with the highest degree of differentiation, *MYCN*-amplified NBs and NBs with mesenchymal features were the most undifferentiated subtypes, containing tumor cells with features of early neuroblasts and bridge cells that remarkably expand the potential pools of malignant cells [44].

Subsequently, Gartlgruber et al. [87] identified four super-enhancers (SEs) epigenetic subtypes and regulatory cell identities in NB. In addition to three ADRN signatures, 60 NB tumors and 25 cell lines employed in this study were shown to form a common cluster with MES signature. However, differently from NB cell lines, which are clearly distinct in two groups with very high or low MES scores, tumors showed a continuous dependency on MES signature, which is suggestive of cellular heterogeneity that may mask malignant MES clones in NB tumors that are instead enriched during cell culture. This work also disclosed that the MES subtype shares cellular identity with multipotent SCPs and can experimentally be triggered by oncogenic *RAS* activation, which indicates that specific mutations can revert neuronal differentiation programs and induce stem cell-like features. Furthermore, mapping of the in-house bulk NB RNA-seq data [44] from these epigenetic subtypes onto the sc-transcriptomes of mouse developing AGs [42] revealed phenotypic similarities with distinct cell populations at different stages of differentiation, from SCPs to early neuroblast/chromaffin cells, demonstrating a considerable NB cellular heterogeneity [87]. These findings are in line with the observation by Kameneva et al. [45] of NBs with highly heterogeneous composition, including malignant cells of both adrenergic and mesenchymal lineages. Among the identified epigenetic subtypes of NB, the MES subtype distinctly overlapped SCPs, suggesting a high degree of phenotypic and molecular similarities [87].

In summary, these studies highlight that, besides tumors with clear ADRN signatures, a cluster of adrenergic NBs with increased MES features aligned with embryonic mesenchymal populations and included cells with traits of SCPs, bridge cells, and connecting progenitors in addition to early neuroblasts and immature chromaffin cells [44, 45, 87]. These results disclosed that adrenal NBs with MES features contain a broad spectrum of undifferentiated cell subtypes that may undergo impairments and functional perturbations that eventually lead to malignant transformation at early stages of development, when sympatho-adrenal and mesenchymal lineages bifurcate from bipotent progenitors (Fig. 3, 2^nd^ split).

The possible contribution of mesodermal lineage derivatives in the formation of NB was another aspect investigated [45]. The adrenal cortex originates from an early mesenchymal progenitor, sharing a transcriptional signature with early kidney progenitor cells, and its development proceeds in tight coordination with NC-derived cells. For this reason, this study has compared sc-gene expression signatures of these developing cells with NB transcription signatures from patients with different survival prognosis [45].

The presence of embryonic adrenocortical and kidney signatures in tumors were associated with poor outcome in non-*MYCN*-amplified NB cases, while no outcome difference was detected in *MYCN*-amplified NBs. This suggested a role of local microenvironment in the modulation of non-*MYCN*-amplified tumors by mesenchymal, cortical, endothelial and kidney cell types, whereas the microenvironment dependencies of tumors with *MYCN* amplification resulted distinct [45].

Very recent insights emerged from the latest sn-transcriptome analysis that compared NB tumor cells from patients classified in different clinical risk groups and stages with healthy cells from postnatal human and mouse AGs [74]. The analysis was extended to previously published sc-sequencing datasets from other NB tumors from the TARGET and from the SEQC NB RNA-seq datasets [77] and from mouse [42] and human [43] embryonic sympatho-adrenal derivatives to provide a better understanding of the transcriptional basis of the clinical heterogeneity in NB [74]. The authors identified a new cluster of TRKB+ cholinergic progenitor cells unique in human postnatal AGs sharing a specific gene signature with a cluster of undifferentiated cells of mesenchymal nature, including biomarkers of migratory/progenitor cell states and a significantly high expression of the *NTRK2* gene, encoding TRKB, which is enriched in high-risk NBs [74]. Moreover, the gene signature of these mesenchymal cells matched to lower patient survival probability and older age-at-diagnosis when assessed in a large cohort of NB patients from NB RNA-seq datasets. Conversely, the transcriptional profile of low-risk NB showed a noradrenergic signature matching the profile of postnatal chromaffin cells as well as of embryonic sympathoblast and chromaffin populations. These results suggest that NBs could also originate from alterations occurring during postnatal development in these cholinergic progenitors. Finally, analyses of cell populations revealed different gene expression programs for worse and better survival in correlation with age at diagnosis, highlighting that the cellular identities and the composition of human NB tumors reflect clinical heterogeneity and outcome [74].

## Core regulatory circuitries and super-enhancers in neuroblastoma

Core regulatory circuitries (CRCs) constitute a network that controls cell gene expression programs, while super-enhancers (SEs) drive expression of genes playing prominent roles in both physiology and cancer.

SEs are functional constituent units that concentrate multiple developmental signaling pathways at key pluripotency genes in embryonic stem cells and derivatives and confer enhanced responsiveness to signaling of their associated genes. Cancer cells frequently acquire SEs at genes that promote tumorigenesis, and these genes are especially sensitive to perturbation of oncogenic signaling pathways. SEs thus provide a program for signaling pathways to regulate genes that control cell identity during development and tumorigenesis [88, 89].

It is now well established that SE-associated TF networks underlie lineage identity [90], but the role of these SEs in intra-tumoral heterogeneity has also emerged and it is still under investigation.

### Intra-tumoral heterogeneity of neuroblastoma: adrenergic and mesenchymal cell identities are associated with regulatory subtypes

Although most established NB-derived cell lines morphologically appear to be composed of a homogeneous cell population, since the 80s some cell lines like SK-N-SH and SK-N-BE (2)-C were described to exhibit different morphological phenotypes. Particularly, SK-N-SH cells were shown to harbor an S-type and an N-type able to undergo a spontaneous bidirectional interconversion [91, 92].

The recent characterization of 33 NB cell lines by transcriptional and epigenetic profiling by van Groningen et al. [32] has identified two predominant cell identities that explain the different phenotypes in NB cells: an undifferentiated MES cell type and a committed ADRN cell type. MES and ADRN cells were found to be the main constituents of intra-tumor heterogeneity of NB, not only in NB cell lines but also in NB specimens, and can spontaneously interconvert into each other by altering their transcriptional states through an epigenetic reprogramming mechanism [32, 33].

This characterization conducted by mRNA profiling and ChIP-seq analysis of H3K27ac, a well characterized marker for active enhancers [93], led to the discovery of two SE-associated TF networks that epigenetically define and shape the MES and the ADRN cell identities and the intra-tumoral heterogeneity and control the gene expression program of NB [32].

Core regulatory circuitry transcription factors (CRC TFs) self-regulate by binding to their own SEs and cross-regulate the expression of other CRC TFs by binding to their SEs, creating a feed-forward loop. These strong interactions drive elevated levels of CRC TFs expression and induce expression of downstream gene expression programs, thereby determining the variety of phenotypes of each cell type and imposing lineage identity [32].

**Fig. 4:**
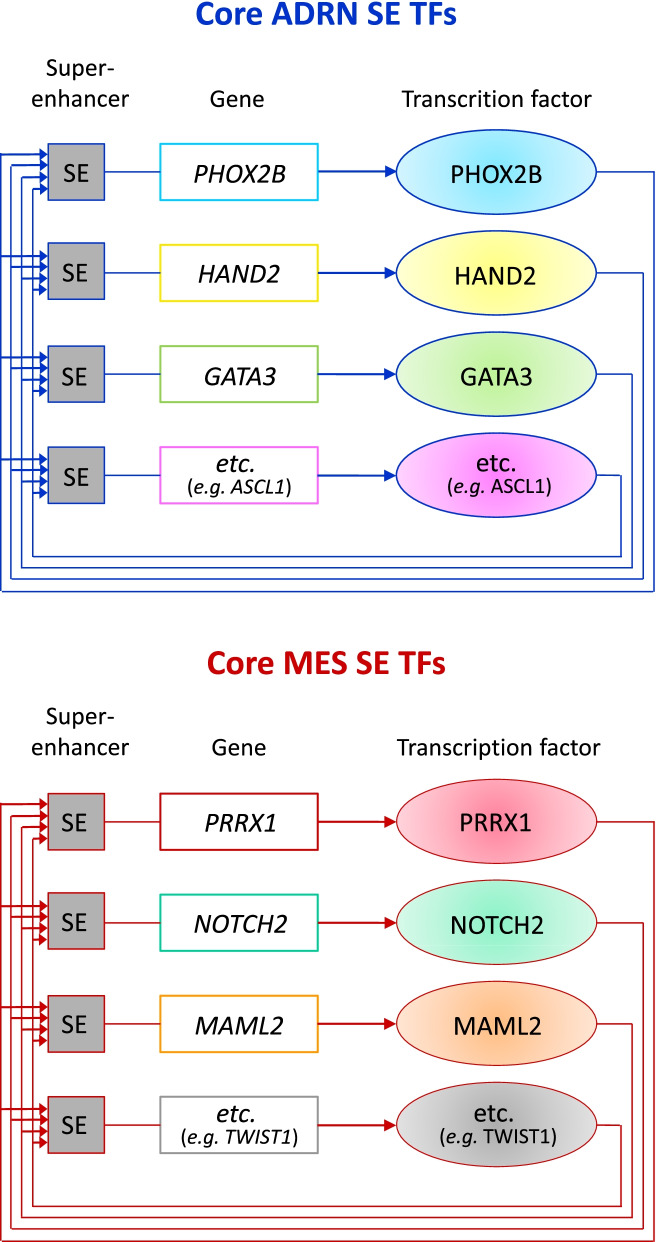
Lineage-specific CRC and SE-associated TFs in MES and ADRN NB cells. Lineage-specific CRCs of ADRN and MES cell identities in NB are composed of a set of SE-associated lineage TFs that bind to each other and induce a powerful feed-forward loop. Illustration modified from Tim J. B. van Groningen, 2020 [94]

ChIP-seq analysis of isogenic pairs of MES and ADRN NB cell lines revealed the distinct SE landscape associated with the expression of lineage TFs of the CRC for each NB cell type (Fig. 4). The identified MES-specific CRC consists of 20 TFs, including PRRX1 and SNAI2, TWIST1, TWIST2, NOTCH2, NOTCH3, and MAML2, while the ADRN CRC includes 18 TFs, among which PHOX2A, PHOX2B, HAND1, HAND2, ASCL1, GATA2, GATA3, and DBH and TH enzymes for catecholamine biosynthesis, all involved in the specification of the sympathetic nervous system [31]. Remarkably, trans-differentiation from the ADRN to the MES identity was shown in vitro following overexpression of PRRX1, demonstrating PRRX1 ability to reprogram the transcriptional and epigenetic profiles from the ADRN to the MES cell state. These results showed that CRC TFs are potent inducers of lineage identity and that the two SE-associated TF networks mediating lineage control in normal development are involved in the epigenetic control of NB and shape intra-tumoral heterogeneity [33].

Analysis of 122 NBs of all stages revealed the presence of both MES and ADRN mRNA signatures placed in between the MES and ADRN counterparts of the NB cell lines. This underlined that NBs are a mixture of both cell types, for most tumors shifted toward ADRN state, confirming the intra-tumoral heterogeneity of NB. Remarkably, MES cells resulted more chemo-resistant in vitro and were enriched in post-therapy and relapsed tumors, suggesting that the reversion of cell identity and plasticity of NB is likely to be involved in response to therapy [32].

In line with these findings, another study conducted in 25 NB cell lines and in 3 matched primary/relapsed NB tumors identified distinct cell identities and the corresponding CRCs [75].

In NB cell lines a sympathetic noradrenergic identity was defined by a CRC module including PHOX2B, HAND2 and GATA3 transcription factors and TH and/or DBH enzymes.

Functional experiments demonstrated dependency of noradrenergic NB on PHOX2B. An NCC-like identity resulted directed by a CRC module including AP-1 and PRRX1 TFs and FOS and JUN family members. Moreover, a mixed-type identity was also identified and treatment with chemotherapeutic agents in NB cell lines ended with an enrichment of NCC-like cells. To find out correlations between TF expression of each CRC module identified in patient tumors, the authors used expression data from a large set of primary NBs (SEQC NB dataset) [95]. Most primary tumors showed expression of TFs from the noradrenergic module and a continuum was observed from low to high values of the NCC-like module. These findings further support the intra-tumoral heterogeneity of cell identity [75], in keeping with the results of the study mentioned above [32]. Nevertheless, NB tumors at relapse were not systematically enriched in NCC-like cells, which supports the concept of plasticity in the reversion of cell identity. This may rely on a switch from adrenergic to NCC-like identity during chemotherapy, and from NCC-like to noradrenergic identity after treatment. Similarly to cell lines, a low expression of the NCC-like module was observed in the majority of *MYCN*-amplified NB tumors, suggesting a role for *MYCN* in downregulation of genes involved in peripheral neuronal differentiation [62, 75].

Regardless of the different terms used to define the distinct cell identities, the results from both studies are consistent, CRC modules overlap, and demonstrate the NB intra-tumoral heterogeneity and the ability of each cell type to interconvert spontaneously.

### Role of transcription factors in reprogramming neuroblastoma cell states

The mechanisms of trans-differentiation are still largely unknown, but the discovery of cell types that differ in phenotype, SEs, and TFs belonging to specific CRCs, led studies to further investigating the gene pathways involved in cell reprogramming [96].

The spontaneous and bi-directional interconversion of cell state observed in NB cell lines relies on the epigenetic control by CRC TFs and confers intra-tumoral heterogeneity to NB [33].

Within a large set of NB cell lines used to investigate the trans-differentiation between the ADRN and MES cell states, the SH-SY5Y and the SH-EP2 cell lines, both deriving from the SK-N-SH [97], characterized by an ADRN and a MES phenotype, respectively, and the SK-N-BE (2)-C with a ADRN signature were determined as the most appropriate and informative in vitro models.

In a more recent study, it was shown that the inducible overexpression of the TF PRRX1 shifted the super-enhancer and gene expression profiles of SK-N-BE (2)-C to a MES state by induction of the MES marker SNAI2 and repression of the ADRN markers PHOX2B and DBH, demonstrating that PRRX1 is able to reprogram the transcriptional and epigenetic landscapes of ADRN cells towards a MES state [33]. However, shRNA-mediated silencing or CRISPR/Cas9-mediated knock-out of *PRRX1* in three MES cell lines did not trigger ADRN differentiation [32]. These results indicate that the depletion of PRRX1 alone is not sufficient to induce a reverse switch and that other TFs of MES CRC may have a crucial role in maintenance of MES phenotype.

CRC of MES cells includes NOTCH2, NOTCH3, and MAML2, that are TFs of the NOTCH pathway, which imposes cell identity switches during development [98].

To test the reprogramming potential of NOTCH pathway in the trans-differentiation of ADRN to MES lineage identity, an inducible NOTCH-IC (NOTCH-intracellular domain) transgene system for all three gene paralogues was employed in SH-SY5Y cells [33]. NOTCH-IC transgenes induced expression of HES1, confirming activation of the NOTCH pathway, and the expression of each NOTCH paralogue induced expression of the MES marker proteins FN1 and SNAI2. Conversely, protein levels of ADRN markers PHOX2A, PHOX2B, and DBH were decreased more efficiently by the NOTCH3-IC [33]. These findings indicated that NOTCH-IC activates an endogenous feed-forward loop among NOTCH receptors leading to transcriptional and epigenetic reprogramming of ADRN cells to a MES state [33]. This transcriptional feed-forward circuitry of NOTCH family TFs, which amplifies the NOTCH signaling levels, can explain the steady transition between ADRN and MES. The kinetics of reprogramming revealed a stepwise transition from the ADRN to a MES state, characterized by an initial repression of ADRN genes such as *PHOX2A*, *PHOX2B*, *DBH*, *GATA2,* and *GATA3*, followed by induction of MES gene expression such as *FN1* and *YAP1* occurring within 1 week.

To investigate whether NB tumors remained oncogenic after massive trans-differentiation to the MES phenotype, inducible NOTCH3-IC SY5Y cells were inoculated in immunodeficient mice. ADRN-to-MES reprogramming was demonstrated to be tumorigenic also in vivo, showing that MES and ADRN cells are equally oncogenic. Tumors with NOTCH3-IC expression showed downregulation of the ADRN signature and induction of the MES signature comparable to the effect of NOTCH3-IC in vitro [33].

Overall, this study demonstrated that reprogramming by NOTCH signaling induced genome-wide remodeling of the H3K27ac landscape and drove a switch from ADRN SEs to MES SEs and that, once established, the NOTCH feed-forward loop was able to maintain the induced MES state.

This transition was mediated by NOTCH3-IC, which downregulated expression of 12 out of 18 expressed TFs from the ADRN CRC, including PHOX2A, PHOX2B, and ASCL1, and induced genome-wide remodeling of lineage-associated SEs. These findings suggest that tumor progression is supported by an interdependency of phenotypically distinct tumor cells [33].

In another study, a CRISPR/Cas9 approach in the SH-SY5Y cell line was used to knock-out TFs of the ADRN CRC and to evaluate the critical TFs that control MES-to-ADRN remodeling [99]. Ablation of *PHOX2A* or *PHOX2B* did not induce morphological, phenotypic, or transcriptional changes in the cells. Conversely, GATA3 knock-out in SH-SY5Y cells led to a MES-like phenotype, to an increased invasion and migration properties, and to a strong reduction of expression of ADRN CRC TFs, including PHOX2A, PHOX2B, and HAND2 [99]. Bulk RNA-seq analysis showed that GATA3-depleted SH-SY5Y cells showed a MES transcriptomic profile clustering with the profile of the MES-type SH-EP cell line of common origin. Consistently, these cells also exhibited an SE landscape similar to that of SH-EP cells. These results, therefore, demonstrated a substantial plasticity of the noradrenergic SH-SY5Y cell line that was able to trans-differentiate toward a MES identity after GATA3 knock-out. Moreover, this work showed that MES NB cells of three different models, including the *GATA3* ^−^/^−^ SH-SY5Y cells, re-expressed noradrenergic and neuroendocrine markers and trans-differentiated their identity towards an ADRN state when engrafted in mice [99]. The authors suggested that the high plasticity exhibited by NB cells also in vivo may be due to interactions between tumor cells and other cell populations of the microenvironment that may provide a powerful pressure from MES–to-ADRN state and thereby may influence the tumor phenotype and progression [99].

Very recently, a compelling review on the plasticity of NB has been published, defining the cell identity transition as Noradrenergic-to-Mesenchymal Transition (NMT) and, vice versa, Mesenchymal-to-Noradrenergic Transition (MNT), addressing some issues on the relationship between cell states and chemoresistance [100]. The authors showed that NMT was reminiscent of, but different from, EMT since they were driven by different inducers. EMT was controlled by effectors such as SNAI1/2, ZEB1/2, TWIST1/2 leading to the expression of E-cadherin and claudins, while in NB NMT was promoted by PRRX1 or NOTCH3-IC overexpression and GATA3 ablation leading to the expression of mesenchymal markers such as vimentin and fibronectin [100].

Finally, the most recent study by Westerhout and co-workers [101] investigated the role of MES cells in relapse development and in response to targeted ALK inhibitors (ALKi), providing very important clues to functional interactions with a specific SE that can trigger the epigenetic regulation of the *ALK* gene*.* At the *ALK* locus, a strong SE was identified which was bound by the core ADRN TFs and, consistently, it was present in ADRN cell lines but absent in MES cells [101]. Remarkably, in an inducible model where NOTCH3-IC drives an ADRN-to-MES transition in SH-SY5Y cells, carrying a p.F1174L ALK mutation [10–12], it was demonstrated that in vitro reprogramming erased the *ALK* SE, silenced either wild-type or mutant *ALK* expression, and, consequently, conferred resistance to ALKi [101]. Also in vivo, this model confirmed that ADRN-to-MES reprogrammed cells can escape from ALK targeted therapy [101] using different ALKi [102]. These findings further support the involvement of cell identity reversion and plasticity in response to therapy, suggesting the need to design MES-specific drugs.

After testing the expression of several genes of the extrinsic apoptosis pathway, like Caspase 8, known to be preferentially expressed in MES cells, the apoptosis inducer TRAIL was shown to efficiently induce apoptosis in MES cells, disclosing that these cells are indeed druggable [101]. By an inducible system, TRAIL was also shown to cause a complete regression of established xenograft tumors in vivo, indicating it may represent a specific drug against MES cells. Using the ALKi Lorlatinib [103], NB tumors formed in vivo by SH-SY5Y xenografts underwent regression, but relapsed after cessation of treatment. Thus, it was tested whether this may be due to a selection of MES cells escaped from the targeted ALKi and, indeed, Lorlatinib and TRAIL combined treatment delayed relapses, laying the bases for combined therapeutic approaches [101]. In this study, it was also demonstrated that MES cells resemble SCPs, sharing with them migratory properties and lack of *ALK* expression. In contrast, ADRN cells resembled committed sympathoblasts that do express *ALK*. These findings suggested that resistance to ALK inhibitors can originate from reiteration of embryonal gene expression programs [101].

### Enhancer invasion and co-occupancy by transcription factors in core regulatory circuitries

TFs of a specific CRC may have distinct roles in shaping cell identity in NB and may allow the definition of critical dependency genes. A study based on an unbiased genome-scale CRISPR-Cas9 approach identified 147 candidate gene dependencies selective for *MYCN*-amplified NB cell lines [104]. A genome-wide ChIP-Seq analysis detected a small number of essential transcription factors such as MYCN, HAND2, ISL1, PHOX2B, GATA3, and TBX2 belonging to the ADRN transcriptional CRC that maintains cell state in MYCN-amplified NB. The authors demonstrated that each of these TFs is able to directly regulate the expression of its own gene as well as of those encoding the other CRC TFs. Consistently, the knockdown of only one member of the CRC induced a decrease of the expression of several other members. As high-level expression of MYC or MYCN was also found in *MYCN* non-amplified NB, a similar set of TFs was hypothesized to form a CRC also in these tumors [104].

As mentioned before, the mechanism of enhancer hijacking is used by NB cancer cells to activate c-*MYC*/*MYCN* expression, [80, 81]. This mechanism occurs when, following structural rearrangements or translocations, distal regulatory elements are brought in proximity to the transcriptional unit of other genes, thereby enhancing their expression. In the case of *MYCN*, ectopic enhancers or CRC-driven SEs were found to be juxtaposed to MYCN amplicon that lacks local enhancers, which highlights the relevance of the CRC in *MYCN* regulation [81]. A common set of ADRN CRC-driven enhancers was detected uniquely in *MYCN* expressing NB cells, which indicates that *MYCN* expression is regulated by CRC TFs, even in the context of gene amplification [81]. These discoveries could mechanistically explain the observation in a previous study that genetic depletion of CRC TFs represses *MYCN* expression even in *MYCN*-amplified cells [104].

This ADRN transcriptional CRC that maintains cell state in *MYCN*-amplified cells can also control an extended regulatory network of genes contributing to initiation and maintenance of the transformed phenotype in MYCN-amplified NB. To disable the CRC, a combination of inhibitors of BRD4 and CDK7, encoded by target genes of the CRC, was tested in vitro and in vivo inducing a rapid downregulation of CRC TF gene expression. This study defined a set of critical dependency genes in *MYCN*-amplified NB that are essential for cell state, growth, and survival and lead to an enhanced vulnerability of these cells to a combinatorial targeting of key proteins mediating transcriptional initiation and elongation [104].

Evidence demonstrated that when expressed at deregulated levels, MYCN invades enhancers to drive tumor-specific processes in NB [23]. Specifically, deregulated MYCN dominates the active cis-regulatory landscape of NB to enforce both proliferation through promoter binding and de-differentiation through enhancer invasion. This mechanism occurs when elevated levels of MYCN protein occupy the enhancers of target genes (enhancer invasion), frequently together with other TFs (co-occupancy) [23].

The co-occupancy of MYCN and other TFs at enhancer sites was evaluated by ChIP-seq analysis on ADRN and MES NB cell lines. It was demonstrated that the clustered non-canonical E-boxes at enhancers invaded by MYCN were proximally occupied by the lineage-specific TF TWIST1 (MES CRC) and HAND2 (ADRN CRC), thereby contributing to drive oncogenic enhancer-driven transcription of target genes. ﻿Interestingly, TWIST1, a transcriptional target of MYCN, was found to be required for MYCN-dependent proliferation, hence, co-occupancy can be considered a deregulated MYCN-specific NB dependency.﻿ Conversely, loss of MYCN led to a global reduction in transcription, more evident at the MYCN target genes with the highest enhancer occupancy [23]. In another study, ChIP-seq and RNA-seq analyses revealed that ASCL1, a fundamental TF involved in neuronal commitment and differentiation, is directly regulated by two TFs having an oncogenic role in NB, namely LMO1 and MYCN [105, 106]. It also emerged that ASCL1 binds to the associated enhancers and regulates the expression of the ADRN neuroblastoma CRC members PHOX2B, HAND2, GATA3, TBX2, and ISL [32, 75, 104], in concert with LMO1 and MYCN, and it is necessary for NB cell growth and arrest of differentiation [107].

In case of *MYCN* amplification/overexpression following an enhancer hijacking process [80, 81], the downstream transcriptional upregulation exerted by MYCN was demonstrated to occur by a mechanism of enhancer invasion [23], through which MYCN reinforces the gene expression program of the entire ADRN CRC in NB [107].

SEs are characterized at structural level by clustering of multiple constituent enhancers in close genomic proximity to each other that interact with the basal transcription machinery at promoters of the target genes [108].

**Fig. 5:**
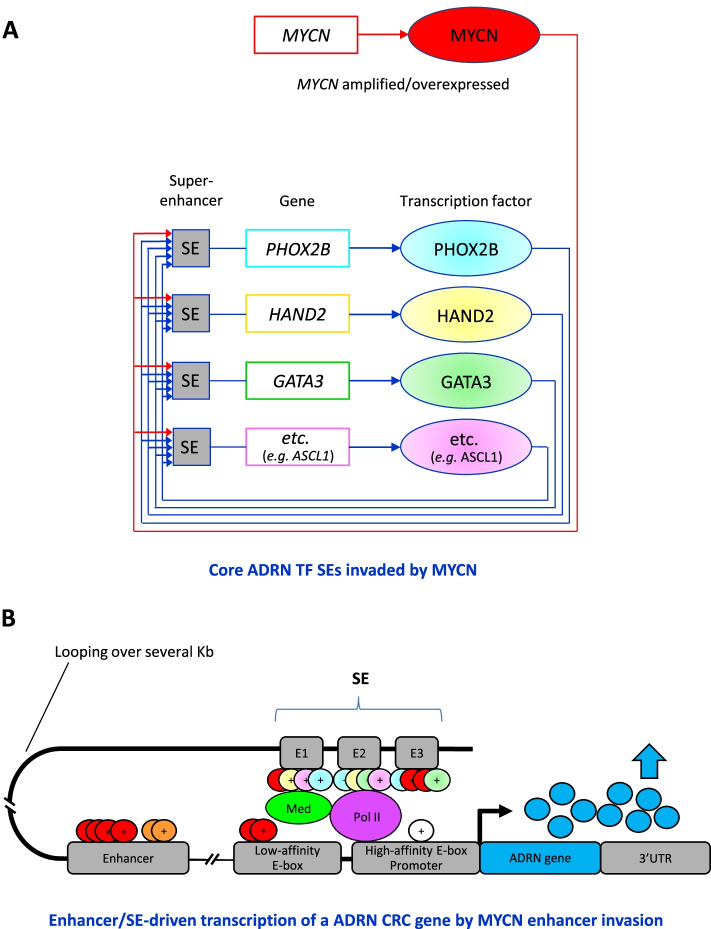
Enhancer/super-enhancer-driven transcription of ADRN CRC genes by MYCN invasion. **A** Schematic representation of the ADRN CRC composed of a set of super-enhancer (SE)-associated lineage TFs (e.g. PHOX2B, GATA3, HAND2, etc.) invaded by MYCN (red arrows) following *MYCN* amplification/overexpression. Each TF (ovals) binds its own SE and each of the other SEs belonging to the CRC, thus inducing a powerful feed-forward loop in all the other genes. **B** Structural drawing of the molecular interactions within the transcriptional unit of a gene belonging to the ADNR CRC (cyan box). The white circle indicates a general TF bound to the promoter site to activate transcription. Starting from the 5′ end, an SE of the ADRN CRC (as indicated in the upper part A) consists of a clustering of E-boxes in close genomic proximity to each other (e.g. three in this figure) bound by all TFs taking part in the ADRN CRC (soft color circles) and invaded by MYCN (red solid circles) when it is amplified/overexpressed. Thanks to the recruitment of the mediator (Med) complex, an SE can interact with the basal transcription machinery and RNA polymerase II (Pol II) at the promoter of the target gene through a looping process to drive its transcription. In addition, overexpressed MYCN can bind a low-affinity E-box or an enhancer of the target gene, thus strongly activating its transcription (thick arrow) and increasing the amount of the gene products (cyan solid circles). The enhancer can be co-occupied by additional TFs together with MYCN (e.g. HAND2 or TWIST1, yellow-orange circles). Invasion of regulatory elements by increased levels of MYCN lead to an enhancer/SE-driven transcription of the target gene. TFs that activate transcription are indicated with “+” within circles. Illustration slightly modified from Perri et al., 2021 [5]

We summarized and illustrated in Fig. 5 how amplified/overexpressed MYCN is able to invade either enhancers or SEs in ADRN CRC, leading to enhancer/SE-driven transcription activation of genes belonging to the ADRN CRC [5]. The oncogenic collaboration of MYCN with other TFs at enhancer sites demarcated a set of developmental genes important for NB tumorigenesis and extremely sensitive to perturbations of the invader, MYCN, and of the MYCN-occupied gene expression program. The disruption of the MYCN enhancer regulatory axis may be considered as a promising therapeutic strategy in NB [23].

### Distinct super-enhancer signatures match neuroblastoma risk subtypes

To determine how distinct cell identities may impact NB tumor development, progression and relapse, a recent study was conducted in NB cell lines and in 60 NB patient samples, covering the different clinical and molecular subtypes [87]. Using genome-wide profiles of H3K27ac, the authors identified four major SE-driven epigenetic subtypes and their corresponding transcriptional CRC networks [87].

The identified SE target genes were found to be associated with: (i) cellular migration and EMT; (ii) neuronal developmental processes; (iii) transcriptional regulation and signaling; (iv) regulation of cell differentiation; and (v) metabolic processes. These SE target genes included well-established NB-related genes such as *CCND1* and *ZNF423,* and a group of TFs and other relevant genes regulated by the ADRN regulatory network such as *PHOX2B*, *ALK*, *MYCN*, *HAND2, MEIS2*, *LMO1*, *GATA2–3* or by the MES network such as *PRXX1*, and *MAML3, RARB*, *NFKB* and *FOSL2* [87] as previously described [32, 75, 106, 107].

Based on their distinct SE signatures, three out of the four identified SE subtypes were characterized by ADRN specific signatures that matched NB tumor samples from different clinical groups: MYCN-amplified high-risk (MYCN_SE_), MYCN non-amplified high-risk (MNA-HR_SE_), and MYCN non-amplified low-risk (MNA-LR_SE_). The fourth subtype, exhibiting mesenchymal characteristics (MES_SE_) and sharing cellular identity with multipotent SCPs, resulted enriched in relapsed disease [87]. Therefore, the authors compared primary and relapse samples from the same patients and found that the MES_SE_ signature activity was strongly increased and the MES specific CRC TFs significantly overexpressed in relapsed and metastatic tumors compared to the matched primary tumors [87]. Notably, as all NB cell lines derive from advanced stage tumors with an aggressive phenotype, MYCN_SE_ and MNA-HR_SE_ as well as MES_SE_ signatures were also found in cell lines, as previously reported [32, 75], while the MNA-LR_SE_ signature was present only in tumor samples from low-risk patients. Importantly, MNA-LR NBs retained SE activity and SE target gene expression of cell identity and neuronal differentiation genes that are mostly suppressed in the other epigenetic subtypes. However, some NB tumors exhibited multiple epigenetic signatures, suggesting that epigenetic subtype cells also contribute to intra-tumoral heterogeneity. Finally, when evaluated SE-directed transcriptomic signatures for association with clinical outcome, samples with high MYCN_SE_ expression activity were associated to the worst overall survival of patients, while samples with the strongest MNA-LR_SE_ expression activity were associated with the most favorable outcome. MNA-HR_SE_ and MES_SE_ expression profiles were associated with similar intermediate outcomes [87]. Besides the main subtype-specific modules, the authors identified highly specific CRC TF subtype modules such as the MYCN subtype including MYCN, TWIST1, SOX11, and TBX2 and the ADRN MNA-LR module including ZNF423, FOXO3, MEIS2, and CREB5.

Taken together, these findings extend the identification of CRCs defined in NB cell lines to those defined in NB tumors that comprise four SE-defined epigenetic NB subtypes.

Consistently with a subtype-specific regulatory activity, binding sites of CRC TFs of the MES subtype had generally more accessible chromatin in MES cells compared to ADRN cells, and vice versa. Moreover, the knockdown effect of these master regulators correlated with the corresponding MES or ADRN TF activity, therefore MES cells resulted more sensitive to the knockdown of MES-specific CRC TFs, such as NFKB2, RUNX1, and RARB, and ADRN cells were more sensitive to the knockdown of adrenergic master regulators, such as *PHOX2B*, *HAND2,* and *GATA3*. These results support the notion that cell survival in the epigenetic subtypes is dependent on subtype-specific CRC networks [87].

Of note, the activity of CRC TFs also revealed a substantial overlap between ADRN and MES SE-subtypes, suggesting the possibility of common regulatory mechanisms. Knockdown profiles of 17 NB cell lines were compared with the profiles of 546 other cancer cells [109] to identify subtype-independent essential genes. Among SE target genes that are not CRC TFs, *CCND1*, encoding the cyclin D1 and often dysregulated in various types of cancer, turned out to be essential across NB cell lines, including both MES and ADRN NB cells, and to be significantly overexpressed across NB cells compared to other cancer cells. These experiments revealed a dependency of both MES and ADRN NB cells on CCND1 [87].

## Conclusions

The studies herein described have led to the identification of the sympatho-adrenal cell populations involved in the physiologic development and of the genes specifying each cell type, which are relevant to NB oncogenesis.

**Fig. 6:**
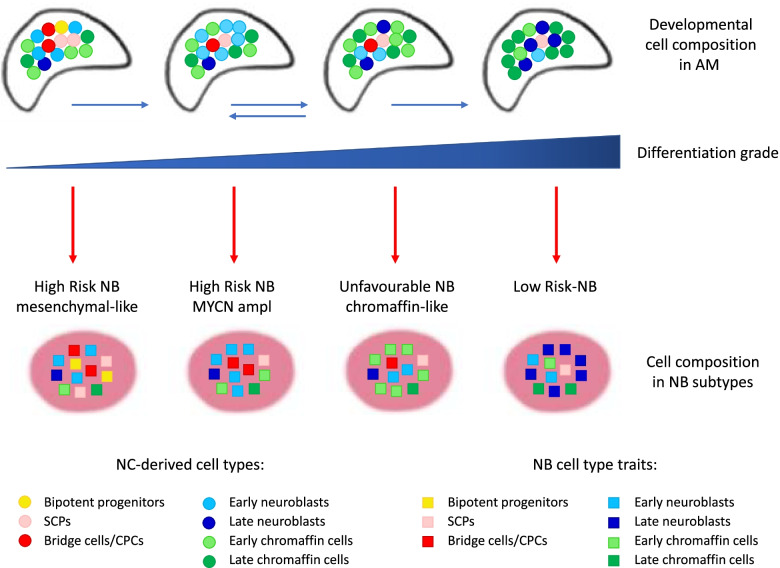
Scheme of the cell composition in human developing AM and in NB risk subtypes. Physiologic developmental cell composition of AM during consecutive differentiation stages (upper part, blue arrows). Genetic alterations or epigenetic perturbations at different stages of AM differentiation may hit different cell types promoting cell selection and malignant transformation (red arrows, lower part). Illustration modified from Hermann Rohrer, 2021 [86]

The different cell types that may be involved in tumor transformation according to the most recent studies are reported in Fig. 6.

The developmental cell composition of the human AM has been dissected at different time points along differentiation trajectories, revealing the presence of many cell types with increasing degrees of differentiation: from progenitors to poorly differentiated neuroblast/chromaffin cells passing through transient cells up to a predominance of differentiated chromaffin cells and some differentiated neuroblasts that compose the mature AM (Fig. 6, upper part, blue arrows).

The prolonged time of cell transitions observed in human sympatho-adrenal development increases the risk for each cell type to acquire genetic alterations or to undergo epigenetic signal perturbations promoting NB formation at different levels. Particularly susceptible to genomic and epigenetic alterations are the NC-derived cells having a high transcriptional activity, whereby chromatin is open, such as bipotent progenitors and SCPs, transient SCP-derived cells (bridge cells, CPCs), immature neuroblasts, and chromaffin cells with low degrees of differentiation or with an enhanced proliferative state (cycling cells) that they maintain following malignant transformation (Fig. 6).

Most consensus results have shown that adrenal NB cancer cells transcriptionally resemble the embryonic adrenal neuroblasts, emphasizing the role of immature sympathetic cells or their closest progenitors in tumor transformation [44, 45, 72, 77]. However, it has also been observed that a fraction of NB tumor cells co-aligned with chromaffin cells [45]. As intra-adrenal sympathoblasts undergo a second transition into local chromaffin cells and, chromaffin cells through a reverse transition may also convert into sympathoblasts [45] (Fig. 3, 2^nd^ transition), the alignment of some NB cells with chromaffin cells highlights that the expression patterns of neuroblasts and chromaffin cells are highly dynamic and show overlapping expression programs during this transition. Therefore, it is reasonable that NB may also arise from a chromaffin cell state that assumes the transcriptional state of sympathoblasts during malignant transformation (Fig. 6, upper part, bi-directional blue arrows).

The comparison of NB cells from tumors of patients classified into risk subgroups with the normal sympatho-adrenal populations has led to cell type assignments differentially associated with NB phenotype severity and overall survival rates. Results have shown that bridge cells and early neuroblasts match NB tumor cells with mesenchymal features, which represent the most undifferentiated subtypes and are associated with high-risk NBs [32, 33, 75] (Fig. 6, lower part). Mesenchymal-like high-risk NBs contain a broad spectrum of undifferentiated cell subtypes that presumably maintain bipotent progenitor cell features (Fig. 6), reminiscent of the second split that separates the sympatho-adrenal and the mesenchymal lineages (Fig. 3). Mesenchymal-like high-risk NBs may undergo transcriptional or epigenetic impairments at early stages of development, holding the highest malignant potential (Fig. 6). Immature or poorly differentiated neuroblasts have been found to match NB tumor cells of the high-risk group, especially *MYCN*-amplified NB cells, and to be associated with poor prognosis, while mature or highly differentiated neuroblasts have been shown to match NB cells of the low-risk group and *MYCN*-nonamplified tumor cells and to be associated with good prognosis [44, 45, 72, 86] (Fig. 6). However, some embryonic chromaffin cells have been aligned with tumors with unfavorable outcomes, suggestive of an immature chromaffin-like NB cell subtype [45] (Fig. 6).

These findings indicate that high-risk tumors derive at early stages of adrenal neuroblasts differentiation trajectory, while low-risk tumors arise at a later time point during development, which reflects the differentiation status of AM development at the time of onset of genetic or epigenetic hits (Fig. 5). Alternative modes of NB initiation and progression may involve the de-differentiation of normal, precancerous, and tumor cells [86], especially in *MYCN*-amplified NBs [44]. We can hypothesize that a de-differentiation process may be induced especially in *MYCN*-amplified tumors thanks to the *MYCN* enhanced expression that represses target genes promoting differentiation and activates genes maintaining pluripotency [22, 29, 110–113].

Very recently, further intriguing findings on the transcriptional basis of NB heterogeneity have revealed that high-risk NBs resemble a new TRKB+ cholinergic progenitor population identified in human postnatal AG [74]. Therefore, it is proposed that favorable NBs might originate from embryonic developmental errors when SCPs differentiate to chromaffin and neuroblast cells, whereas part of unfavorable NBs could arise from alterations occurring during postnatal development when TRKB+ cholinergic progenitors repopulate chromaffin cells postnatally [74].

Taken together, the new knowledge on the developmental origin of NB, herein reviewed, points out that NB cancer cells are associated with reduced differentiation, high proliferative capacity, and maintenance of stemness properties typical of developmental cell populations. These features can also be present in the reservoir of immature cells of the differentiated tissue of origin during the postnatal period.

From the molecular point of view, the discovery of specific CRCs, SEs, and associated TFs in NB has provided fundamental clues for a deeper understanding of cell identity gene expression programs in normal development and in tumorigenesis.

Evidence proved that NB is composed of two SE-associated differentiation states that identify two epigenetically distinct identities i.e., committed adrenergic (ADRN) and undifferentiated mesenchymal (MES) cell types [32]. These cell types may interconvert into each other, which explains the cellular and the intra-tumoral heterogeneity of NB and discloses a high plasticity of NB that relies on transcriptional and epigenetic reprogramming [32, 33, 75, 87, 100, 104, 107].

Based on transcriptional signatures, most adrenal NB primary tumors show a predominant expression of TFs from the ADRN module, though clustering of adrenergic tumors with increased expression of the MES module do exist. As NBs with MES features are enriched in metastatic, post-therapy and relapsed tumors, which are more resistant to chemotherapy, transcriptional and epigenetic analyses become powerful tools to distinguish the epigenetic landscapes of NB tumors and to define among the alternative reprogrammed transcriptional states which is repressed or inducted. This knowledge provides the basis for the design of targeted therapies specifically directed to MES cells for preventing relapse [32, 33] by either inducing specific cell death or a shift to an ADRN identity. Other authors have hypothesized that chemotherapy may transiently promote a switch from ADR to MES state and that reversion from MES to ADRN may occur when the treatment pressure decreases. This should suggest the importance of targeting both cell types during cancer treatments [75, 99, 100]. In this regard, very recently, it has been proven that ADRN-to-MES reprogrammed NB cells can escape from ALK targeted therapy, disclosing that NB cancer cells with an undifferentiated phenotype can mediate resistance to ALK inhibitors [101]. This research has also demonstrated that TRAIL treatment specifically targets MES cells and induces apoptosis and that in combination with an ALK inhibitor it delays relapse, offering a perspective for cell type specific treatment or combined therapeutic approaches [101].

Although in some studies the signature of medullary SCPs and late SCPs has been associated with favorable prognosis or a better survival [45, 72], in the most recent study based on SE-defined subtypes of NBs [87] and NB models in vitro and in vivo [101], MES cells have been found to share cellular identity with multipotent SCPs. In view of the epigenetic reprogramming that NB cells may undergo, the presence of SCP traits, found in either NBs with favorable outcome or in MES undifferentiated NB tumors, may indicate different cell states adopted by multipotent precursors upon malignant transformation.

The identification of two distinct SE landscapes, ADRN and MES, involved in the epigenetic control of NB through the expression of lineage TFs and specifier genes for each cell-type CRC, also provides many molecular targets for therapeutic approaches.

The identified ADRN-specific CRC includes important TFs like MYCN, PHOX2A, PHOX2B, ASCL1, HAND2, GATA2, GATA3, MEIS2, LMO1, TBX2, ISL1 and the tyrosine kinase receptor ALK, and DBH and TH enzymes involved in the metabolism of catecholamines. The MES-specific CRC includes the TFs PRRX1, TWIST1, SNAI2, MAML3, RUNX1, NFKB, AP-1 family members (JUN and FOS) and the retinoic acid receptor beta, RARB [32, 33, 75, 87, 107] (Fig. 4).

In addition, a recent study conducted in NB cell lines and in NB tumor samples from different clinical and molecular subtypes has identified four major SE-driven epigenetic subtypes and their corresponding transcriptional CRC networks, highlighting their impact on NB tumor development, progression, and relapse [87].

The discovery of specific CRC TFs for each cell type and subtypes and interconnected CRC genes has also allowed the definition of the critical dependency genes that are essential for cell state, growth, and survival in NB. Of note, the identification of a highly specific CRC TFs and subtypes with selective dependency that controls and maintains the cell state in MYCN-amplified NB cells [104]. In addition, it has been shown that MES cells are more sensitive to the knockdown of MES-specific CRC-TF and ADRN cells are more sensitive to the knockdown of ADRN-specific CRC-TF regulators [87].

All these studies provide a better understanding of the molecular factors that control NB phenotypic plasticity and demonstrate the involvement of SEs at key genes that promote tumorigenesis and are extremely sensitive to disturbance of oncogenic signaling pathways.

The dependency on transcriptional activity of a set of TFs and their target genes involved in initiation and maintenance of the transformed phenotype represents a vulnerable point for NB cells that may be exploited for therapeutic interventions. RNAi molecules or protein small inhibitors can be leveraged to interfere/block the oncogenic functions of crucial CRC TFs and their downstream targets within specific or combinatorial targeting approaches.

This knowledge on the complex gene circuitries that are deregulated in NB, open new perspectives into the design of promising therapeutic strategies based on the transcriptome profiles to remodel aberrant regulatory networks from a dysregulated expression, which blocks differentiation and enhances proliferation, toward a controlled expression that prompts the most differentiated state.

Since there is strong evidence that TFs are involved in resistance to cytotoxic drugs [114–116], novel therapies aimed at targeting CRC TFs may be relevant to make tumor cells more sensitive to chemotherapy and may represent a beneficial therapeutic strategy to improve the outcome of high-risk NB patients with refractory disease or chemo-resistant relapse and to lay the basis for patient-tailored therapies.

Combinatorial strategies that integrate RNAi-mediated silencing approaches with conventional chemotherapy and immunotherapy may lead to clinical applications to obtain a more effective therapeutic response.

In this overview of the most recent findings that extend our understanding about the NC progeny involved in the oncogenesis of NB, we have attempted to provide updated and consistent information about the developing cell types that may undergo malignant transformation and to give an overall picture. However, some controversial conclusions of studies based on similar approaches [40, 43, 47, 48] highlight how a rigorous cell type annotation based on the expression of specific markers, an adequate sample size, and comparable computational analyses are critical points in this research field.

In the near future, we expect that additional investigations will provide new insights and fill the gap of knowledge about the processes that lead developing cells towards tumor cell transformation to definitively shed light upon the development of NB.

## Data Availability

Not applicable.

## Abbreviations

ADRN: Adrenergic
AM: Adrenal medulla
Cas9: CRISPR associate protein 9
ChIP-seq: Chromatin Immunoprecipitation Sequencing
CPCs: Connecting Progenitor Cells
CRCs: Core regulatory circuitries
Cre-loxP: Cre recombinase-mediated recombination between LoxP sites
CRISPR: Clustered Regularly Interspaced Short Palindromic Repeats
E10.5, E11.5, E13.5: embryonic developmental mouse timeline expressed in days
EMT: Epithelial-to-mesenchymal transition
FISH: Fluorescent In Situ Hybridization
MES: Mesenchymal
NB: Neuroblastoma
NC: Neural crest
NCCs: Neural crest cells
scRNA-seq: Single-cell RNA sequencing
SCPs: Schwann Cell Precursors
SA: Sympatho-adrenal
SE/s: Super-enhancer/s
SNPCs: Sympathetic Neural Progenitor Cells
SRG: Suprarenal sympathetic ganglion
TF/s: Transcription factor/s
Wpc: Weeks post conception
Alk/ALK: Anaplastic lymphoma kinase
AP-1: Activator Protein-1
Ascll/ASCL1: Achaete-Scute Family BHLH Transcription Factor 1
ATRX: Alpha-thalassemia/mental retardation, X-linked
BMPs: Bone Morphogenetic Proteins
BRD4: Bromodomain Containing 4
Cart/CART: Cocaine- and amphetamine-regulated transcript
Cartpt/CARTPT: CART prepropeptide
Cas9: CRISPR associated protein 9
CCND1: Cyclin D1
CDK7: Cyclin dependent kinase 7
CDKN1C: Cyclin dependent kinase inhibitor 1C
Chga/CHGA: Chromogranin A
Chgb/CHGB: Chromogranin B
COL1A1: Collagen type I alpha 1 chain
CREB5: cAMP responsive element binding protein 5
Dbh/DBH: Dopamine beta-hydroxylase
EBF1: Early B cell factor 1
ERBB3: Erb-b2 receptor tyrosine kinase 3
ERBB4: Erb-b2 receptor tyrosine kinase 4
EXOC4: Exocyst complex component 4
FGF: Fibroblast growth factor
FN1: Fibronectin 1
Fos/FOS: Fos proto-oncogene
FOSL2: FOS like 2
FOXD: Forkhead Box D3
FOXO3: Forkhead box O3
GAP43: Growth Associated Protein 43
Gata2/GATA2: GATA binding protein 2
Gata3/GATA3: GATA binding protein 3
Gfap/GFAP: Glial fibrillary acidic protein
H3K27ac: Histone H3 lysine 27 acetylation
Hand2/HAND2: Heart and neural crest derivatives expressed 2
HES1: Hes family bHLH transcription factor 1
Hmx1: H6 family homeobox 1
Htr3a: 5-hydroxytryptamine receptor 3A
IL7: Interleukin 7
INSM1: Insulinoma-associated 1 transcriptional repressor 1
ISL1: Islet LIM homeobox 1
Jun/JUN: Jun proto-oncogene
LIN28B: Lin-28 homolog B
LMO1: LIM domain only 1
MAML: Mastermind like transcriptional coactivator 2
MAML3: Mastermind like transcriptional coactivator 3
MARCH11: Membrane-associated ring finger (C3HC4) 11
Mash1: Alias of Ascl1
MEIS2: Meis homeobox 2
MKI67: Marker of proliferation Ki-67
MYC: MYC proto-oncogene, bHLH transcription factor
Mycn/MYCN: MYCN proto-oncogene, bHLH transcription factor
NEFM: Neurofilament medium chain
Neurog2: Neurogenin 2
NFKB: Nuclear factor kappa-B
NFKB2: Nuclear factor kappa B subunit 2
Notch/NOTCH: Notch receptor 1
NOTCH2: Notch receptor 2
NOTCH3: Notch receptor 3
NOTCH-IC: NOTCH-intracellular domain
NPY: Neuropeptide Y
NRG1: Neoregulin 1
*NTRK1*: Neurotrophic receptor tyrosine kinase 1, encoding TRKA
*NTRK2*: Neurotrophic receptor tyrosine kinase 2, encoding TRKB
Penk/PENK: Proenkephalin
Phox2a/PHOX2A: Paired like homeobox 2A
Phox2b/PHOX2B: Paired like homeobox 2B
Plp1/PLP1: Proteolipid protein 1
PNMT: Phenylethanolamine N-methyltransferase
Prph/PRPH: Peripherin
Prrx1/PRRX1: Paired related homeobox 1
PTPRB: Protein tyrosine phosphatase receptor type B
PTPRC: Protein tyrosine phosphatase receptor type C
RARB: Retinoic acid receptor beta
RAS: Rat sarcoma
Ret/RET: Ret proto-oncogene
RUNX1: RUNX family transcription factor 1
Scg10/SCG10: Alias of Stmn2/STMN2
SDF1: Stromal cell-derived factor alias CXCL12
SNAI2: Snail family transcriptional repressor 2
Sox10/SOX10: SRY-box transcription factor 10
SOX11: SRY-box transcription factor 11
SOX4: SRY-box transcription factor 4
STMN2: Stathmin 2
SYN3: Synapsin III
TBX2: T-box transcription factor 2
TERT: Telomerase reverse transcriptase
TFAP2B: Transcription factor AP-2 beta
Th/TH: Tyrosine hydroxylase
TOP2A: DNA topoisomerase II alpha
TRAIL: TNF-related apoptosis inducing ligand
*Trk*: Neurotofic receptor kinase
TRKB (encoded by *NTRK2*): Neurotrophic receptor tyrosine kinase 2
TWIST1: Twist family bHLH transcription factor 1
TWIST2: Twist family bHLH transcription factor 2
VGF: VGF nerve growth factor inducible
WNT: Wingless-type MMTV integration site family
YAP1: Yes1 associated transcriptional regulator
ZNF423: Zinc finger protein 423
